# Association between attention-deficit/hyperactivity disorder symptom severity and white matter integrity moderated by in-scanner head motion

**DOI:** 10.1038/s41398-022-02117-3

**Published:** 2022-10-06

**Authors:** Sabine Dziemian, Zofia Barańczuk-Turska, Nicolas Langer

**Affiliations:** 1 Department of Methods of Plasticity Research, Institute of Psychology, University of Zurich, CH-8050 Zurich, Switzerland; 2 University Research Priority Program (URPP) Dynamic of Healthy Aging, University of Zurich, CH-8050 Zurich, Switzerland; 3 Neuroscience Center Zurich (ZNZ), CH-8057 Zurich, Switzerland; 4 Center for Reproducible Science (CRS), University of Zurich, CH-8001 Zurich, Switzerland; 5 Institute of Mathematics, University of Zurich, CH-8057 Zurich, Switzerland

**Keywords:** Neuroscience, Biomarkers, Psychology

## Abstract

Attention-deficit/hyperactivity disorder (ADHD) is a common and debilitating neurodevelopmental disorder associated with various negative life impacts. The manifestation of ADHD is very heterogeneous, and previous investigations on neuroanatomical alterations in ADHD have yielded inconsistent results. We investigated the mediating effect of in-scanner head motion and ADHD hyperactivity severity on motion-corrected fractional anisotropy (FA) using diffusion tensor imaging in the currently largest sample (*n* = 739) of medication-naïve children and adolescents (age range 5–22 years). We used automated tractography to examine whole-brain and mean FA of the tracts most frequently reported in ADHD; corpus callosum forceps major and forceps minor, left and right superior-longitudinal fasciculus, and left and right corticospinal tract (CST). Associations between FA and hyperactivity severity appeared when in-scanner head motion was not accounted for as mediator. However, causal mediation analysis revealed that these effects are fully mediated through in-scanner head motion for whole-brain FA, the corpus callosum forceps minor, and left superior-longitudinal fasciculus. Direct effect of hyperactivity severity on FA was only found for the left CST. This study illustrates the crucial role of in-scanner head motion in the identification of white matter integrity alterations in ADHD and shows how neglecting irremediable motion artifacts causes spurious findings. When the mediating effect of in-scanner head motion on FA is accounted for, an association between hyperactivity severity and FA is only present for the left CST; this may play a crucial role in the manifestation of hyperactivity and impulsivity symptoms in ADHD.

## Introduction

Attention-deficit/hyperactivity disorder (ADHD) is a common neurodevelopmental disorder diagnosed in approximately 7.2% of school-age children [1]. ADHD affects brain development and manifests in persistent impairments of inattention, elevated impulsivity, and hyperactivity [2]. Depending on symptom domination, ADHD is classified into a predominantly inattentive presentation (ADHD-IN), a predominantly hyperactive-impulsive presentation (ADHD-HI), and a combined presentation (ADHD-C) [2]. Besides substantial evidence of negative consequences for personal, social, and academic functioning, the disorder’s etiology remains insufficiently understood [3]. Amongst environmental and biological risk factors [3], alterations in fronto-striatal-cerebellar brain networks are assumed to play a causal role in the pathophysiology of ADHD [4–8]. White matter integrity probed by diffusion-weighted imaging (DWI) bears the potential to reveal white matter tract abnormalities [9].

However, past research on the white matter underpinnings of ADHD has produced discrepant results [5, 6, 10, 11]. Findings in ADHD are inconsistent in both the tracts reported to deviate and the direction of deviation, most commonly quantified by the diffusivity measure of fractional anisotropy (FA) [5, 6, 10, 11].

Previous findings are exceptionally mixed for the corpus callosum (CC), the superior longitudinal fasciculus (SLF), and the corticospinal tract (CST) including the corona radiata (CR), internal capsule (IC), and cerebral peduncle (CP) [5, 12]. Some studies found lower FA compared to controls in children with ADHD in the CC and its subparts [12–21], in the SLF [12, 14, 15, 22–25], and in the CST, including the CR, IC, and CP [9, 12–17, 22, 23, 26–29]. In contrast, other studies either identified higher FA in children with ADHD in these structures [17, 29–34] or could not find any group difference at all (CC (incl. subparts): [18, 22, 27, 35–42]; SLF: [21, 27, 35, 37, 39–42]; CST: [35, 36, 41]). Remarkably, for almost each study reporting a structure with lower FA in ADHD, another study reports the opposite. For a detailed review of previous DWI studies see [10] and Table S1 in Supplement A.

Potential reasons for these discrepancies may be small sample sizes [6], and dissimilar sample demographics (e.g., age range, gender distribution and diagnostic exclusion criteria) [5, 6, 10, 43], medication histories [6, 10, 43], and analysis approaches [5, 6, 10, 11, 43], including statistics [6, 10, 43]. Furthermore, previous studies lacked a consistent representation of ADHD. Some studies used a categorical approach that derived the diagnosis from common classification systems (i.e., DSM-5, ICD-10). Such categorical studies tend to disregard mutually exclusive manifestations of ADHD presentations by focusing only on the overarching category of ADHD (see [38]) and potentially miss distinct underlying mechanisms [34, 44]. In contrast, other studies chose dimensional approaches in line with the research domain criteria (RDoC), focusing on ADHD severity or leveraging behavioral measures and rating scales to characterize the disorder (see [45]). Studies investigating symptom severity of ADHD mostly utilized rating scale designs that do not capture both positive and negative behavioral extremes (e.g., Conners Behavior Rating Scales), which may result in floor effects in the symptom spectrum [46, 47]. Consequently, an important unresolved objective in ADHD research is to demonstrate which approach best reflects the manifestations of ADHD before investigating neuroanatomical alterations.

Another major issue in past research concerns the handling of in-scanner head motion during preprocessing and statistical analysis [6, 10, 48]. While the role of in-scanner head motion has been of great concern in functional MRI studies (e.g., 49), it has been of lesser concern in DWI [10]. In DWI, even small in-scanner head motion profoundly impacts scan quality [50] and subsequently derived neuroanatomical measures, potentially leading to false group differences [12, 48, 51, 52]. In a recent meta-analysis [10] of 25 DWI studies on ADHD, only five studies considered head motion estimates as a confounding factor or ruled out group differences in motion. Yet, these studies mostly failed to find significant group results [10]. Far more strikingly, a previous meta-analysis [6] observed that only half of the studies examined reported correcting for in-scanner head motion at all [6]. Although post hoc correction of in-scanner head motion is widely applied, the correction relies on models of estimated motion which cannot entirely reverse the distorting impact [48]. Consequently, knowledge of motion estimates must be used in all statistical analyses in which differences in motion are expected to mitigate spurious group differences [6, 48, 51, 52]. This is clearly the case in ADHD, of which excessive motion constitutes a characteristic intrinsic to hyperactivity and impulsivity [6, 52–55]. Limiting the sample to participants with little in-scanner head motion may seem plausible, but it imposes an underrepresentation of the full symptom severity of ADHD.

Here, we investigate the causal mediation effect of in-scanner head motion and ADHD on whole-brain and tract-wise FA of six tracts previously most commonly reported in relation to ADHD; forceps minor of CC, forceps major of CC, left and right CST, and left and right SLF. Prior to the causal mediation analysis, we compared two approaches to identify which ADHD representation best explains the measured data: using an ADHD rating scale for a full-spectrum dimensional representation of hyperactivity severity [46] and the DSM-5 diagnosis for a categorical representation [2]. To the best of our knowledge, this is the first study that has analyzed the causal mediation effect of in-scanner head motion on FA in a large pediatric sample.

## Methods and materials

### Participants

Participants were included from the Healthy Brain Network (1st–7th release), an ongoing open data acquisition initiative by the Child Mind Institute [56]. All participants underwent a comprehensive screening for any mental disorder. Upon indication, an extensive assessment was performed for specific mental disorders with corresponding supplementary behavioral tests. All diagnoses were given according to the DSM-5 on consensus by multiple licensed clinicians [2]. Prior to participation, legal guardians or participants of legal age provided written informed consent. Study approval was given by the Chesapeake Institutional Review Board.

Participants were included if they received an ADHD diagnosis or had no diagnosis of any mental disorder (i.e., controls). Participants underwent acquisition of structural T1-weighted imaging and diffusion tensor imaging (DTI). All participants included were right-handed, defined as an Edinburgh Handedness Inventory (EHI) total score above 40 [57, 58], and had a full-scale IQ above 69, measured using the Wechsler Intelligence Scale for Children (WISC-V), Wechsler Abbreviated Scale of Intelligence (WASI), or Wechsler Adult Intelligence Scale (WAIS) according to the age of the participant. Importantly, all participants included in this study were medication-naïve, eliminating any past or current influence of medication on neuroanatomy [36, 59]. Specific exclusion criteria for participants with ADHD were current or past history of schizophrenia spectrum and other psychotic disorders, neurocognitive disorders (e.g., epilepsy), borderline intellectual functioning, and intellectual disability. Individuals with an unspecified or other specified ADHD diagnosis (*n* = 65) or an ADHD-HI diagnosis (*n* = 30) were excluded due to underrepresentation and overall rarity of ADHD-HI in the global population [60]. Specific exclusion criteria for the control group were any current or past history of psychological or neurological disorders.

Participants whose MRI data could not be preprocessed due to severe artifacts or did not pass visual inspection by a rater blind to the demographics and psychological conditions were excluded (see Supplement B). A final sample of 739 participants aged 5–22 years (236 female, mean age = 11.25 ± 3.34) was analyzed. For a full description of demographics, clinical characteristics, and head motion measures, see Table 1. These variables are strongly interconnected, and isolated analyses might be misleading. Therefore, we focused on generalized linear mixed-effects models, which account for cross-correlations between variables. For the interest of the reader, isolated statistical analyses for these measures are included in Supplement C.

**Table 1:** Demographics, clinical characteristics, and head motion measures of the ADHD predominantly inattentive presentation, ADHD combined presentation, and control group.

	ADHD-IN (*n* = 339)	ADHD-C (*n* = 279)	Controls (*n* = 121)
Age, Years, Mean (SD)	12.01 (3.34)	10.38 (3.02)	11.14 (3.55)
Sex, Female, n (%)	119 (35.10%)	61 (21.86%)	56 (46.28%)
IQ, Mean (SD)	98.21 (15.55)	101.15 (15.36)	109.01 (14.37)
Comorbidities, n (%)			
Specific learning disorder	88 (19.13%)	79 (19.75%)	–
Autism spectrum disorder	58 (12.61%)	49 (12.25%)	–
Oppositional defiant disorder	28 (6.09%)	80 (20.00%)	–
Conduct disorder	5 (1.09%)	3 (0.75%)	–
Anxiety disorder	126 (27.39%)	109 (27.25%)	–
Major depressive disorder	37 (8.04%)	17 (4.25%)	–
Other mental disorder	111 (24.13%)	66 (16.50%)	–
SWAN, Raw Score, Mean (SD)			
Inattention	1.14 (0.89)	1.26 (0.85)	−0.42 (1.14)
Hyperactivity	0.14 (0.86)	1.13 (0.81)	−0.57 (1.10)
Total	0.64 (0.69)	1.19 (0.72)	−0.50 (1.02)
Relative Head Motion, mm, Mean (SD)	0.44 (0.36)	0.56 (0.48)	0.50 (0.38)

*ADHD* attention-deficit/hyperactivity disorder, *ADHD-IN* ADHD predominantly inattentive presentation, *ADHD-C* ADHD combined presentation, *SWAN* Strengths and Weaknesses Assessment of Normal Behavior Rating Scale for ADHD.

Clinical diagnoses were given according to the Diagnostic and Statistical Manual of Mental Disorders (DSM-5).

### Variables decoding ADHD

**Fig. 1:**
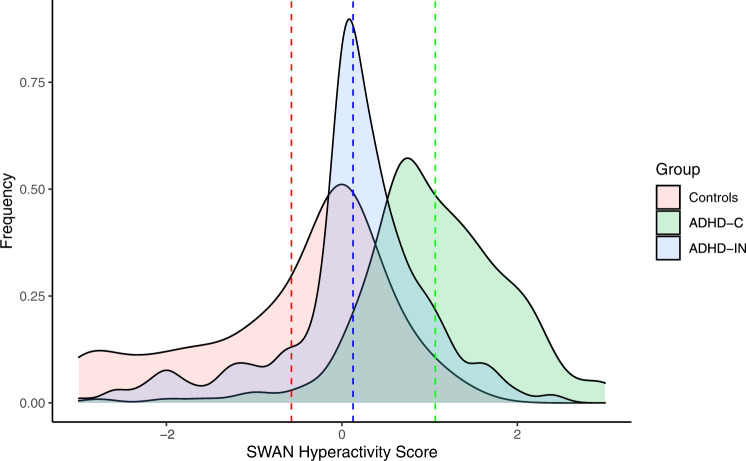
Distribution of SWAN Hyperactivity score by study group. Dashed line: group mean; x- axis: −3: very low; −2: low; −1: slightly lower; 0: average; 1: slightly higher; 2: high; 3: very high. ADHD, attention-deficit/hyperactivity disorder; ADHD-IN, ADHD predominantly inattentive presentation; ADHD-C, ADHD combined presentation; SWAN, Strengths and Weaknesses Assessment of Normal Behavior Rating Scale for ADHD.

The ADHD category was defined by the DSM-5 diagnosis [2]. ADHD symptom severity was assessed using the Strengths and Weaknesses Assessment of Normal Behavior Rating Scale for ADHD (SWAN) [46]. This scale is sensitive to extremes of both high and low hyperactivity and impulsivity (SWAN-HY) and inattention (SWAN-IN). In contrast to other rating scales, which focus on the presence of deficits, the SWAN rating scale prevents floor effects at positive extremes in the normal population and thus preserves the full variability of potential symptom severity [46, 47]. The SWAN-HY score registers behaviors related to excessive motion, which we hypothesize to be expressed in larger in-scanner head motion. Thus, the SWAN-HY score was used as a dimensional measure of hyperactivity severity for the entire sample (Fig. 1). For a visualization of the remaining SWAN scores, see Supplement D.

### MRI data acquisition

DTI and T1-weighted scans were recorded at three sites in the larger New York area. See Alexander et al. 2017 for a full scanning protocol [56] and Supplement E for detailed descriptions of site-specific scanning parameters. One point of particular relevance for this study is that no equipment was used to restrict in-scanner head motion at any of the scanning sites, which is a common procedure in pediatric neuroimaging.

### MRI preprocessing

DTI data were preprocessed based on the recommended diffusion parameter estimation with Gibbs and noise removal (DESIGNER) pipeline [61] using FMRIB Software Library (FSL) version 6.0.4 [62]. A detailed description is provided in Supplement F. The preprocessing code is made available at: https://github.com/sdziem/DTIPreprocessingPipeline.

Briefly, DTI scans were denoised [63] and corrected for Gibbs artifacts [64]. Susceptibility-induced off-resonance field distortion correction was omitted in favor of including a larger sample for whom echo-planar image scans with reversed phase-encode blips were unavailable [65]. For corresponding validation of omitting this correction on tract-wise mean FA, see Supplement G. Next, we applied the FSL Brain Extraction Tool (BET) using an FA threshold of 0.1 [66]. Eddy current-induced distortions were removed using eddy_cuda, which also corrects in-scanner head motion and slice-wise and multiband group outliers [50, 67, 68]. Eddy_cuda additionally performs slice-to-volume correction, which accounts for movements occurring within a volume instead of between volumes. This version outperforms previous implementations and further limits the impact of remaining distortions on subsequently extracted diffusivity measures [50, 67, 68]. Importantly, although we chose state-of-the-art movement correction, residual head movement effects remained because movement can only be estimated, and the ground truth remains unknown. Residual artifacts influence subsequently derived measures of white matter integrity [10, 48, 51]. Therefore, we estimated in-scanner head motion with eddy_quad (quality assessment for DMRI, FSL version 6.0.3) [69] for subsequent causal mediation analysis. In-scanner head motion was calculated as the average relative displacement between volumes in mm. Excessive in-scanner head motion was not applied as an exclusion criterion to preserve full variability in the sample, in whom motion is regarded as a characteristic intrinsic to hyperactivity and impulsivity [52–55]. To confirm that our results are not driven by a few outliers with high in-scanner head motion, we conducted a sensitivity analysis excluding participants with in-scanner head motion equal to or above 2 mm (compare [23, 38, 70, 71]) and confirmed similar results for all statistical analyses (see Supplement K).

Preprocessing continued with outlier detection and robust estimation of MRI parameter [72], tensor fitting, and extraction of diffusivity measures with weighted linear least squares estimation [73–75].

### Tractography

We used the deterministic streamline-tracking algorithm Automating fiber-tract quantification (AFQ, version 1.1) [76–78] to extract objective and reliable diffusion properties quantifying white matter integrity [76, 79, 80].

First, a T1-weighted scan, which was co-registered to the DTI scan, was used to set anatomical regions of interest as seeds for tractography. Second, whole-brain tractography was computed with the default FA threshold of 0.2, an FA mask threshold of 0.3 [76], and an adapted angle threshold of 35° to account for the young range [81–83]. Third, tracts were segmented if they ran through two waypoints in the co-registered T1-weighted scan that define distinct anatomical features of the tract based on a WM atlas [84]. Fourth, fiber tracts were refined by examining the likelihood of a fiber belonging to the given tract from fiber tract probability maps [85]. Next, fiber tracts were cleaned, and outliers within the tract removed using 4 standard deviations from the mean tract length and 5 standard deviations in distance from the tract core [76]. Each fiber was sampled at 100 equidistant segments using the Mahalanobis distance from the given fiber segment to the core along the tract [76]. Finally, FA was calculated for each segment of the tract with a weighted sum of the corresponding fibers determined by the probability of the fiber belonging to the tract [76].

**Fig. 2:**
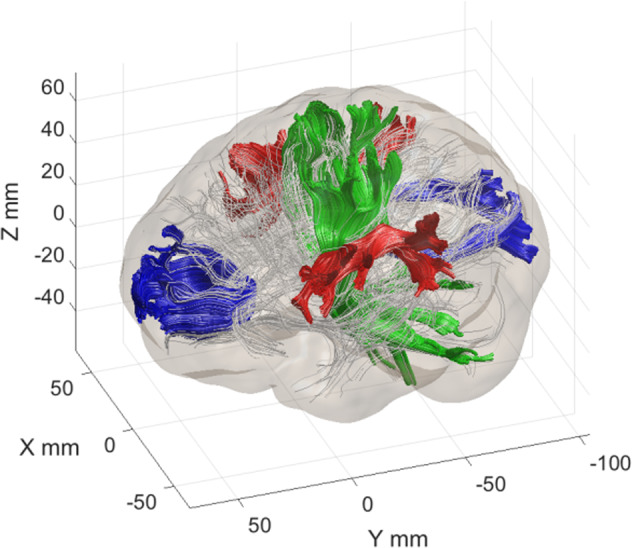
Illustration of anatomical location of regions of interest. Corpus callosum forceps minor and major (blue), left and right corticospinal tract (green), left and right superior longitudinal fasciculus (red), and whole-brain white matter tracts (thin gray lines).

We obtained FA tract profiles for 100 equidistant segments for each participant and tract of interest. From these profiles, we calculated for each participant (a) whole-brain mean FA based on all profiles of all identifiable tracts and (b) a tract-wise mean FA by averaging the FA tract profile for each of the following six tracts: the CC forceps minor and major, the left and right CST, and the left and right SLF (see Fig. 2). Hence, we obtained an average FA value for each tract of interest of each participant, thereby yielding reliable quantifications of participants’ white matter integrity [86, 87]. These were used for subsequent statistical analysis.

### Statistical analysis

All statistical analyses were conducted in R version 4.1.1. Formulas are described using the Wilkinson notation.

#### Model comparison using categorical and dimensional representation of ADHD

First, we identified the best generalized linear mixed-effects model for explaining the data. We used both a categorical and dimensional representation of ADHD on whole-brain FA to identify the most explanatory model for both approaches. The base model contained whole-brain FA (continuous) as dependent variable, in-scanner motion (continuous) as variable of interest and age (continuous), sex (categorical: male, female), IQ (continuous), and acquisition site (categorical: three sites) as confounding factors. The acquisition site was included to account for different scanning sites (compare [12, 41]). The categorical analysis compared the base model to extended models that included the ADHD category (categorical: ADHD-IN, ADHD-C, No ADHD) as a fixed effect and an interaction effect between the ADHD category and in-scanner head motion. The same procedure was applied for the dimensional analysis with the SWAN-HY score (continuous). To compare the models, we excluded participants with an ADHD-HI diagnosis from the dimensional analysis as well. Nested models were compared using an ANOVA (Supplement H). To further determine which model better explains the given data, we directly compared the categorical and the equivalent dimensional models with the same number of predictors using the Akaike information criterion (AIC) and the Bayesian information criterion (BIC).

#### Causal mediation analysis

**Fig. 3:**
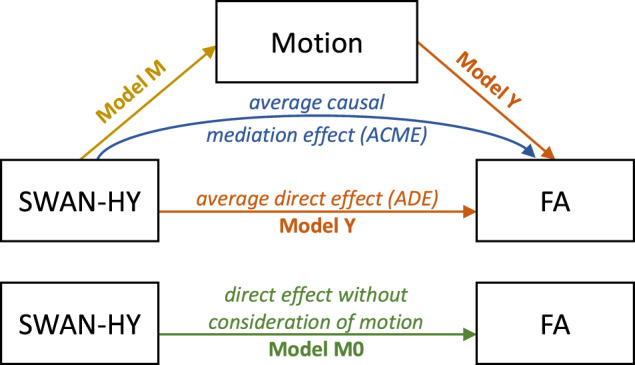
Causal mediation analysis design. Model Y (orange) captures the average direct effect (ADE) of SWAN-HY on FA. Model M (yellow) reflects the effect of SWAN-HY on motion regardless of FA. The indirect effect of SWAH-HY on FA through the mediation of motion is the average causal mediation effect (ACME) (blue). Model M0 (green) captures the relationship of SWAN-HY on FA disregarding any effect of motion. SWAN-HY, Strengths and Weaknesses Assessment of Normal Behavior Rating Scale for ADHD Hyperactivity Score; FA, fractional anisotropy.

We used a causal mediation analysis [88] to investigate whether differences in whole-brain FA and tract-wise FA can be explained by a mediating effect of in-scanner head motion rather than the disorder (see Fig. 3). Based on the selected model (here the dimensional model), Model M0 to assess the direct effect of SWAN-HY on whole-brain and tract-wise mean FA disregarding the effect of in-scanner head motion was defined as

**Model M0:** FA ~ Age + Sex + IQ + Site + SWAN-HY.

Model M to assess the effect of SWAN-HY on in-scanner head motion was defined as

**Model M:** Motion ~ Age + Sex + IQ + Site + SWAN-HY.

Model Y, which comprises the effect of SWAN-HY and the effect of in-scanner head motion on FA, was defined as

**Model Y:** FA ~ Age + Sex + IQ + Site + Motion + SWAN-HY.

Models M and Y were used as input for causal mediation analysis. Figure 3 depicts the mediation relation analyzed in this study. The causal mediation analysis was calculated using the R package for causal mediation analysis with nonparametric bootstrap for confidence intervals and 5000 Monte Carlo draws [88]. Effect sizes for the causal mediation analysis are reported as portions of variance [89]. Because these measures are not squared but are results of arithmetic operations, they can be negative, indicating suppression effects [89, 90]. Prior to the causal mediation analysis, we confirmed a main effect of SWAN-HY in either Model M0 or M (Table 2), which is a premise for consecutive causal mediation analysis [91, 92]. In particular, the main effect of SWAN-HY on motion shows how ADHD symptomatology is associated with an increase in-scanner head motion.

**Table 2:** Summary of regressions of Models M0 and M as premise for causal mediation analysis using the dimensional model.

Structure	Model M0: SWAN-HY → FA	Model M: SWAN-HY → Motion
Whole-Brain FA	*β* = −0.0017	*β* = 0.0577
	*p* = 0.015*	*p* = 0.012*
	CI = [−0.00311, −0.00033]	CI = [0.01259, 0.10284]
	*r*^*2*^_*SF*_ = 0.00814	*r*^*2*^_*SM*_ = 0.00867
CC Forceps Minor	*β* = −0.0033	*β* = 0.0557
	*p* = 0.018*	*p* = 0.016*
	CI = [−0.00595, −0.00056]	CI = [0.01039, 0.10103]
	*r*^*2*^_*SF*_ = 0.00774	*r*^*2*^_*SM*_ = 0.00804
CC Forceps Major	*β* = −0.0006	*β* = 0.0520
	*p* = 0.790	*p* = 0.029*
	CI = [−0.00492, 0.00375]	CI = [0.00538, 0.09872]
	*r*^*2*^_*SF*_ = 0.00011	*r*^*2*^_*SM*_ = 0.00721
Left CST	*β* = −0.0032	*β* = 0.0583
	*p* = 0.025*	*p* = 0.013*
	CI = [−0.00602, −0.00041]	CI = [0.01257, 0.10397]
	*r*^*2*^_*SF*_ = 0.00717	*r*^*2*^_*SM*_ = 0.00887
Right CST	*β* = −0.0029	*β* = 0.0548
	*p* = 0.043*	*p* = 0.018*
	CI = [−0.00565, −0.00010]	CI = [0.00952, 0.10003]
	*r*^*2*^_*SF*_ = 0.00581	*r*^*2*^_*SM*_ = 0.00794
Left SLF	*β* = −0.0023	*β* = 0.0634
	*p* = 0.148	*p* = 0.006**
	CI = [−0.00551, 0.00083]	CI = [0.01787, 0.10889]
	*r*^*2*^_*SF*_ = 0.00295	*r*^*2*^_*SM*_ = 0.01042
Right SLF	*β* = 0.0011	*β* = 0.0574
	*p* = 0.494	*p* = 0.013*
	CI = [−0.00199, 0.00412]	CI = [0.01194, 0.10281]
	*r*^*2*^_*SF*_ = 0.00066	*r*^*2*^_*SM*_ = 0.00854

*SWAN-HY* Strengths and Weaknesses Assessment of Normal Behavior Rating Scale for ADHD Hyperactivity Score, *FA* fractional anisotropy, *CC* corpus callosum, *CST* corticospinal tract, *SLF* superior longitudinal fasciculus, *CI* 95% confidence interval, *r*^*2*^_*SF*_ portion of variance in FA explained by SWAN-HY*, r*^*2*^_*SM*_ portion of variance in motion explained by SWAN-HY.

## Results

### Model selection

Nested model comparison using ANOVA (Supplement H) yielded two models for direct comparison:

**Model Cat:** FA ~ Age + Sex + IQ + Site + Motion + ADHD Category

**Model Dim:** FA ~ Age + Sex + IQ + Site + Motion + SWAN-HY

The direct comparison revealed a better model fit for the dimensional model (see Table 3) due to smaller AIC and BIC. Hence, we concluded that the dimensional model is more suitable for explaining the given data in subsequent causal mediation analysis.

**Table 3:** Comparison of measures to assess model fit between categorical (Model Cat) and dimensional model (Model Dim).

	Residual SE	Multiple R^2^	Adjusted R^2^	AIC	BIC
Model Cat	0.01909	0.6905	0.6871	−3687.038	−3641.135
Model Dim	0.01905	0.6912	0.6882	−3690.604	−3649.291

*SE* standard error, *AIC* Akaike information criterion, *BIC* Bayesian information criterion.

### Causal mediation analysis

**Fig. 4:**
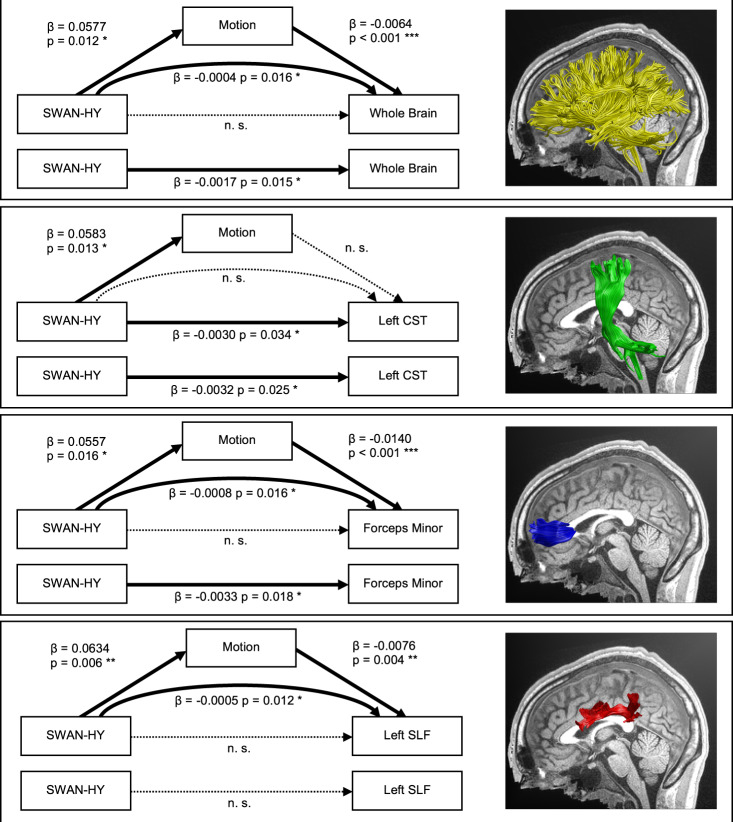
Results of causal mediation analysis with significant effects. SWAN-HY, Strengths and Weaknesses Assessment of Normal Behavior Rating Scale for ADHD Hyperactivity Score; CST, corticospinal tract; SLF, superior longitudinal fasciculus; T1-weighted image: X = −1.

The premise for causal mediation analysis revealed that ADHD symptomatology (i.e., SWAN-HY) is associated with larger in-scanner head motion in all structures analyzed in this study (Table 2). The causal mediation analysis yielded a significant average causal mediation effect (ACME) of in-scanner head motion on whole-brain FA (β = −0.0004; *p* = 0.016; CI_95%_ = [−0.00074, −0.00007]). At tract level, a significant ACME was observed for the CC forceps minor (β = −0.0008; *p* = 0.016; CI_95%_ = [−0.00155, −0.00014]), and the left SLF (β = −0.0005; *p* = 0.012; CI_95%_ = [−0.00103, −0.00008]). An average direct effect (ADE) of SWAN-HY on FA was observed for the left CST (β = −0.0030; *p* = 0.034; CI_95%_ = [−0.00575, −0.00023]) (Table 4 and Fig. 4). Yet, these effects do not survive correction for multiple comparisons when considering a corrected significance level of *p* < 0.0102 using the method suggested by Nyholt (2004) [93] (for calculation of corrected significance level see Supplement M).

**Table 4:** Summary of causal mediation analysis results.

Structure	Average Direct Effect (ADE)	Average Causal Mediation Effect (ACME)
Whole-Brain FA	*β* = −0.0014	*β* = −0.0004
	*p* = 0.057	*p* = 0.016*
	CI = [−0.00270, 0.00003]	CI = [−0.00074, −0.00007]
	*R*^*2*^_*direct*_ = 0.00498	*R*^*2*^_*med*_ = 0.00316
CC Forceps Minor	*β* = −0.0025	*β* = −0.0008
	*p* = 0.060	*p* = 0.016*
	CI = [−0.00501, 0.00014]	CI = [−0.00155, −0.00014]
	*R*^*2*^_*direct*_ = 0.00444	*R*^*2*^_*med*_ = 0.00330
CC Forceps Major	*β* = −0.0002	*β* = −0.0004
	*p* = 0.919	*p* = 0.090
	CI = [−0.00378, 0.00331]	CI = [−0.00102, 0.00004]
	*R*^*2*^_*direct*_ = 0.00001	*R*^*2*^_*med*_ = 0.00009
Left CST	*β* = −0.0030	*β* = −0.0002
	*p* = 0.034*	*p* = 0.166
	CI = [−0.00575, −0.00023]	CI = [−0.00062, 0.00007]
	*R*^*2*^_*direct*_ = 0.00622	*R*^*2*^_*med*_ = 0.00095
Right CST	*β* = −0.0026	*β* = −0.0002
	*p* = 0.058	*p* = 0.086
	CI = [−0.00552, 0.00008]	CI = [−0.00065, 0.00003]
	*R*^*2*^_*direct*_ = 0.00484	*R*^*2*^_*med*_ = 0.00097
Left SLF	*β* = −0.0019	*β* = −0.0005
	*p* = 0.281	*p* = 0.012*
	CI = [−0.00510, 0.00142]	CI = [−0.00103, −0.00008]
	*R*^*2*^_*direct*_ = 0.00185	*R*^*2*^_*med*_ = 0.00110
Right SLF	*β* = 0.0013	*β* = −0.0003
	*p* = 0.386	*p* = 0.072
	CI = [−0.00166, 0.00441]	CI = [−0.00072, 0.00002]
	*R*^*2*^_*direct*_ = 0.00104	*R*^*2*^_*med*_ = −0.00038

*FA* fractional anisotropy, *CC* corpus callosum, *CST* corticospinal tract, *SLF* superior longitudinal fasciculus, *CI* 95% confidence interval, R^2^_med_ portion of variance in FA mediated through Average Causal Mediation Effect (ACME), R^2^_direct_ portion of variance in FA from Average Direct Effect (ADE).

Importantly, Model M0, which disregards the effect of motion, exhibits a main effect of SWAN-HY on whole-brain FA, the CC forceps minor, and left CST (Table 2), but when motion is accounted for in the causal mediation analysis, the effect is truly direct only for the left CST (Table 4). For whole-brain FA and for FA in the CC forceps minor, this effect is fully mediated via in-scanner head motion. The left SLF constitutes a special case in which SWAN-HY was not significant in Model M0 but was in Model M. Here, the influence of SWAN-HY on FA is evident by a significant mediating effect of in-scanner head motion on FA.

### Segment-based analysis

**Fig. 5:**
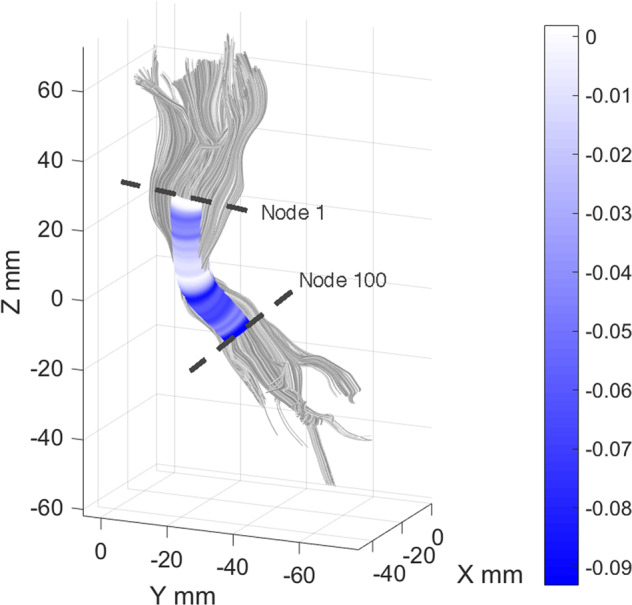
Correlation of fractional anisotropy (FA) for each segment of the corticospinal tract (gray) with Strengths and Weaknesses Assessment of Normal Behavior Rating Scale for ADHD Hyperactivity Score (SWAN-HY) on clipped regions of interest (blue shaded tube). FA values are regressed out for age, sex, IQ, acquisition site and in-scanner head motion. Strongest correlations were observed at caudal segments of the tract. Node, equidistant segment on tract between clipped regions of interest.

In the left CST, we observed an ADE of SWAN-HY on mean FA. This indicates the presence of a relationship between ADHD and FA even when in-scanner head motion is taken into account as a mediator. Figure 5 depicts the correlation of FA values for each segment, regressed out for age, sex, IQ, acquisition site and in-scanner head motion, with the SWAH-HY score. Strongest correlations were observed in caudal areas at the level of the cerebral peduncle.

## Discussion

Previous DTI studies on children with ADHD have reported inconsistent results [5, 6, 10, 11] (see Supplement A). Past research is of limited comparability due to disagreements about categorical vs. dimensional representations of ADHD [34, 44] and the trivialization of incorporating in-scanner head motion as a variable of particular interest [6, 10, 48, 51]. An initial objective of this study was to identify whether white matter integrity is best explained by categorical (DSM-5 diagnosis) or dimensional (SWAN-HY score) representations of ADHD symptoms. We observed that a dimensional approach outperformed the categorical. The major aim of this study was to investigate whether the effect of ADHD symptomatology on FA is mediated by in-scanner head motion with a causal mediation analysis. We investigated the mediation effect of in-scanner head motion on whole-brain and tract-wise FA for the six tracts most commonly reported in ADHD: CC forceps minor and major, left and right CST, and left and right SLF.

### Causal mediation analysis

Prior to the causal mediation analysis, we observed ADHD symptomatology to be associated with lower FA when disregarding in-scanner head motion (Table 2). However, the causal mediation analysis yielded a significant full mediation of in-scanner head motion on whole-brain FA, FA in the CC forceps minor, and in the left SLF. A full mediation indicates no direct effect of SWAN-HY on FA for these structures. Hence, lower FA in these structures is not a direct neural substrate of ADHD. The effects are rather driven by larger in-scanner head motion, as shown by the significant relationship between SWAN-HY and in-scanner head motion (Fig. 4). This relationship can be interpreted as a proxy symptom expression of abnormally high levels of hyperactivity. Furthermore, the effect of in-scanner head motion indicates the presence of remaining motion artifacts in the scans.

In previous literature on the six tracts of interest, only Wu et al. (2017) considered head motion as a confounding factor in their statistical analysis [15], and Nagel et al. (2011) set a maximum head motion threshold of 2 mm for inclusion [23]. However, treating in-scanner head motion as a confounding factor does not reflect its actual role shown by our causal mediation analysis. Accounting for a mediation effect reveals that the actual portion of variance in FA is mediated via motion and is no longer attributable to the SWAN-HY score (Fig. 4). Our results highlight the importance of considering remaining in-scanner head motion artifacts knowing that even minor movement during acquisition can artificially distort FA measures [48, 50, 51], as we also demonstrate in the present study. Therefore, a plausible explanation for previous inconsistent FA findings in ADHD might be the neglect of the role of remaining motion artifacts post correction. Motion is an intrinsic characteristic of hyperactivity, which is elevated in children with ADHD-C and ADHD-HI, compared to ADHD-IN [6, 52–55]. This is substantiated by the present data, in which the extent of in-scanner head motion differs significantly between ADHD presentations (see Table S4). Therefore, we argue that former findings have to be interpreted with caution given the widespread underestimation of in-scanner head motion influence.

In contrast, a direct effect of SWAN-HY on lower FA was observed for the left CST. This supports previous findings of lower FA [9, 12–17, 22, 23, 26–29] in the CST. Importantly, an ADE of ADHD symptomatology on lower FA in the left CST while accounting for mediation effects, discredits spurious observation arising out of an underestimation of in-scanner head motion.

The CST is a projection fiber tract originating at the corona radiata (CR) and extends into the internal capsule (IC) and further into the cerebral peduncle (CP) [94]. The CST is involved in the basic motor system of voluntary movement control [95, 96]. At the level of the CR, the CST receives input from the primary motor cortex, primary somatosensory cortex, supplementary motor area, and dorsal premotor cortex [95]. Traveling via the IC into the midportion of the CP, these inputs are projected via the pontine nuclei to the cerebellum [94]. The research community continuously highlights alterations in the fronto-striatal-cerebellar brain network as entailing the pathophysiology of ADHD [4–8]. The CST subserves the same cortical motor areas as are involved in the fronto-striatal-cerebellar network and projects to the cerebellum at the level of the CP. Our segment-based analysis identified strongest correlations of lower FA with hyperactivity symptoms in caudal segments of the left CST at the level of the CP. This is in line with previous research that has identified this locus as altered in ADHD [9, 26, 28, 29]. Axonal damage to the CP and atypical microstructure of the CST have been associated with poor motor outcomes [97, 98]. Consequently, it is plausible that lower FA in the CP in children with ADHD precipitates concomitant motor deficits considering the structure’s role in motor refinement, motor learning and integration of proprioception. Here, we provide a further account of an impaired motor system in ADHD subserved by poor white matter integrity in the CP of the left CST in children with elevated hyperactivity and impulsivity, evident as reduced ability to suppress in-scanner head motion. Therefore, we suggest that as an additional component to the fronto-striatal-cerebellar network, the left CST should also be considered in the behavioral manifestation of ADHD.

### Limitations

A clear limitation of this study is the lack of an ADHD-HI subgroup in all analyses; this presentation was excluded due to the small sample size. Furthermore, the SWAN-HY score is not a clinical diagnostic measure. High SWAN-HY scores need not imply an underlying ADHD-C or ADHD-HI diagnosis yet are highly correlated with clinical presentations [46].

Another limitation concerns the interpretability of FA and its reflection of white matter integrity. Lower FA is commonly interpreted as resulting from deficient myelination or poorer fiber health, which is often summarized as poorer white matter integrity [99, 100]. However, FA measures are further influenced by the axon diameter, number of axons, and axon branching [101]. Moreover, crossing fibers may lead to lower FA values in a given voxel, which stands in conflict to a conclusion of poorer white matter integrity [101]. Nonetheless, FA reflects the restriction of water diffusion, which is mostly constrained by cell membranes and the degree of myelination. Although further diffusivity measures are necessary to explain the detailed causes of tissue deviations [102–104], FA is an established measure of fiber health and indicative of white matter integrity [99, 100]. Within this study, we focused on the diffusivity measure of FA because this was used in the majority of previous DWI studies investigating white matter alteration in children with ADHD [5, 6, 11, 12]. We investigated whether past FA discrepancies in these studies are due to an underestimation of the impact of in-scanner head motion. Moreover, we aimed at limiting potential error sources in the estimation of FA by using AFQ instead of manual tractography. AFQ is more objective and reliable due to its automatic identification of tract location and is thus also easier to reproduce [76, 105, 106]. Given our large sample size, reliable manual tractography would not have been feasible.

Furthermore, the reported effects would not have survived correction for multiple comparisons when considering a corrected significance level of *p* < 0.0102 using the method suggested by Nyholt (2004) [93]. Thus, future additional studies are required to confirm our findings.

## Conclusions

In light of the disagreement on categorical vs. dimensional representations of ADHD manifestations, we compared these two approaches. We identified the SWAN-HY score as more suitable in comparison to the SWAN-IN score and the clinical DSM-5 diagnosis, and used it in line with the RDoC to identify neuroanatomical underpinnings of ADHD with causal mediation analysis. When disregarding the role of in-scanner head motion, we observed an effect of the SWAN-HY score on FA in several tracts. However, the causal mediation analysis revealed most of these associations to be mediated by in-scanner head motion. Therefore, future investigations should focus on the distorting effect of irremediable motion artifacts in DWI. When accounting for a mediating effect of in-scanner head motion, we identified the left CP of the CST tract as the only structure to be directly influenced by hyperactivity. This finding and the role of the CP in the fronto-striatal-cerebellar neurocircuitry supports its interpretation as a biological substrate of ADHD.

## Supplementary information

Supplemental Material
