# Supplementary material for: Association between attention-deficit/hyperactivity disorder symptom severity and white matter integrity moderated by in-scanner head motion
Source: Transl Psychiatry. 2022 Oct 6;12:434. doi: 10.1038/s41398-022-02117-3 (PMC9537185; doi:10.1038/s41398-022-02117-3)
Supplement: Supplementary file 1 — Supplemental Material [file 41398_2022_2117_MOESM1_ESM.docx]

Supplement

# Review Summary on Fractional Anisotropy Findings in ADHD

**Table S1:** Summary of studies using fractional anisotropy to compare ADHD with controls of selected tracts sorted by structure.

| **Structure** | **Higher FA in ADHD** | **Lower FA in ADHD** |
| --- | --- | --- |
| **Corpus Callosum** | Chou et al., 2007  Silk et al., 2009  Francx et al., 2016 | Cao et al., 2010  Qiu et al., 2011  Chuang et al., 2013  van Ewijk et al., 2014  Langevin et al., 2014  Francx et al., 2016  Pastura et al., 2016  Ameis et al., 2016  Wu et al., 2017  Lin et al., 2020 |
| **Internal Capsule** | Bu et al., 2020 | Chou et al., 2007  Pavuluri et al., 2009  Qiu et al., 2011  Chuang et al., 2013  van Ewijk et al., 2014  Francx et al., 2016  Pastura et al., 2016 |
| **Corticospinal Tract** | Svatkova et al., 2016  Bu et al., 2020 | Ashtari et al., 2005  Hamilton et al., 2008  Bechtel et al., 2009  Kobel et al., 2010  Chuang et al., 2013  van Ewijk et al., 2014  Bu et al., 2020 |
| **SLF** | Silk et al., 2009 | Hamilton et al., 2008  Nagel et al., 2011  van Ewijk et al., 2014  Chiang et al., 2015  Chiang et al., 2016  Pastura et al., 2016  Wu et al., 2017 |
| **Corona Radiata** | Davenport et al., 2010  Tamm et al., 2012 | Pavuluri et al., 2009  Kobel et al., 2010  Qiu et al., 2011  Nagel et al., 2011  Chuang et al., 2013  Francx et al., 2016  Wu et al., 2017 |
| **ILF** | Silk et al., 2009  Svatkova et al., 2016 | Nagel et al., 2011  Chuang et al., 2013  van Ewijk et al., 2014 |
| **Thalamic Radiation** | Peterson et al., 2011  Silk et al., 2009  Tamm et al., 2012  Svatkova et al., 2016 | Pastura et al., 2016  van Ewijk et al., 2014 |
| **Arcuate Fasciculus** | - | Chiang et al., 2016 |
| **Uncinate Fasciculus** | Silk et al., 2009  Tamm et al., 2012 | Nagel et al., 2011 |
| **Cingulate Fasciculus** | Silk et al., 2009 | - |
| **IFOF** | Tamm et al., 2012 | van Ewijk et al., 2014  Pastura et al., 2016 |
| **Cingulum** | Svatkova et al., 2016 | Chiang et al., 2015  Chiang et al., 2016 |
| **External Capsule** | - | Pastura et al., 2016 |

SLF: superior longitudinal fasciculus; IFL: inferior longitudinal fasciculus; IFOF: inferior fronto-occipital fasciculus.

# Quality Control and Sample Exclusions

Not all MRI data initially selected for this study underwent successful preprocessing, for several reasons: (a) incomplete MRI acquisition with missing T1-weighted scan or missing DWI scan, corrupt data, incompatible diffusion directions not covering the whole sphere; (b) incomplete motion correction due to severe artefacts or signal dropouts, incomplete tractography due to remaining artefacts. Furthermore, data were excluded because of (c) insufficient quality rating due to incomplete tractographies, gross artefacts, or misalignments of scans; (d) 1. incomplete psychological assessment, 2. comorbidities of schizophrenia spectrum and other psychotic disorders, neurocognitive disorders (e.g., epilepsy), borderline intellectual functioning, intellectual disability, 3. medication, 4. left-handedness or ambidexterity, 5. full-scale IQ below 70; (e) no ADHD diagnosis but other diagnoses, unspecified ADHD diagnosis, other specified ADHD diagnoses, predominantly hyperactive-impulsive ADHD. See Figure S1 below for a dropout flowchart.


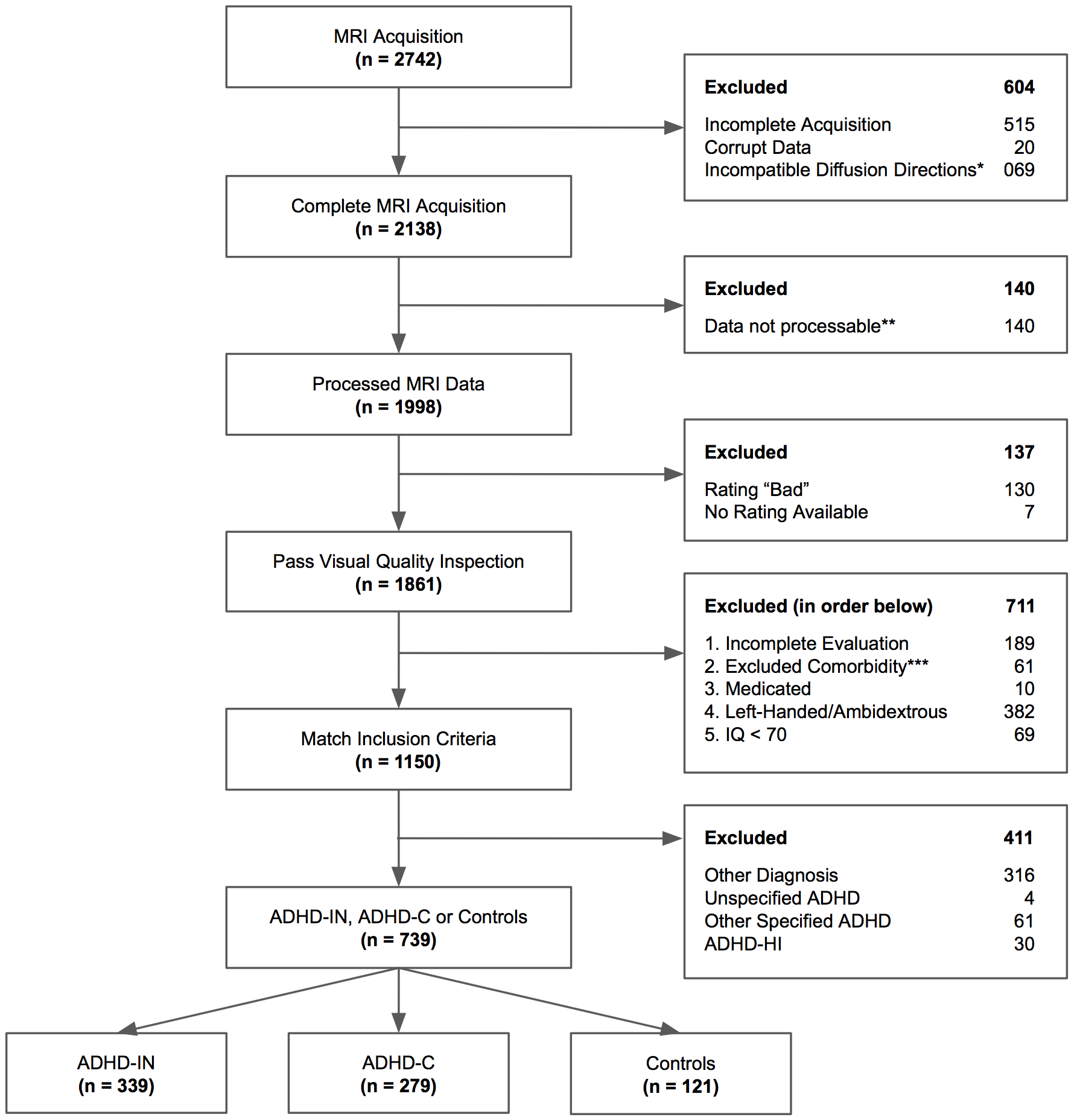


**Figure S1:** Flowchart of data exclusion for final statistical analysis. *Note:* * data not acquired on whole sphere; ** preprocessing according to DESIGNER pipeline, potential reason: unsuccessful correction of excessive head motion, incomplete tractography due to remaining artefacts; *** excluded comorbidities include schizophrenia spectrum and other psychotic disorders, neurocognitive disorders (e.g., epilepsy), borderline intellectual functioning, and intellectual disability.

## MRI Visual Quality Control

To maintain high data quality of all DWI scans, alignments between T1-weighted and DWI scans and whole-brain tractography from AFQ were visually inspected with our tool developed in house for visual inspection. Visual inspection focused on alignments between T1-weighted and DWI scans, full coverage of whole-brain tractography, gross artefacts in position or shape of tracts, and other remaining large artefacts in the DWI scan. The rater was blind to the demographics and diagnosis status of the participants. Data was rated as “good” or “bad”. Only data classified as “good” were included in the subsequent statistical analysis of neuroanatomical measures. An example of good and insufficient quality is provided below.


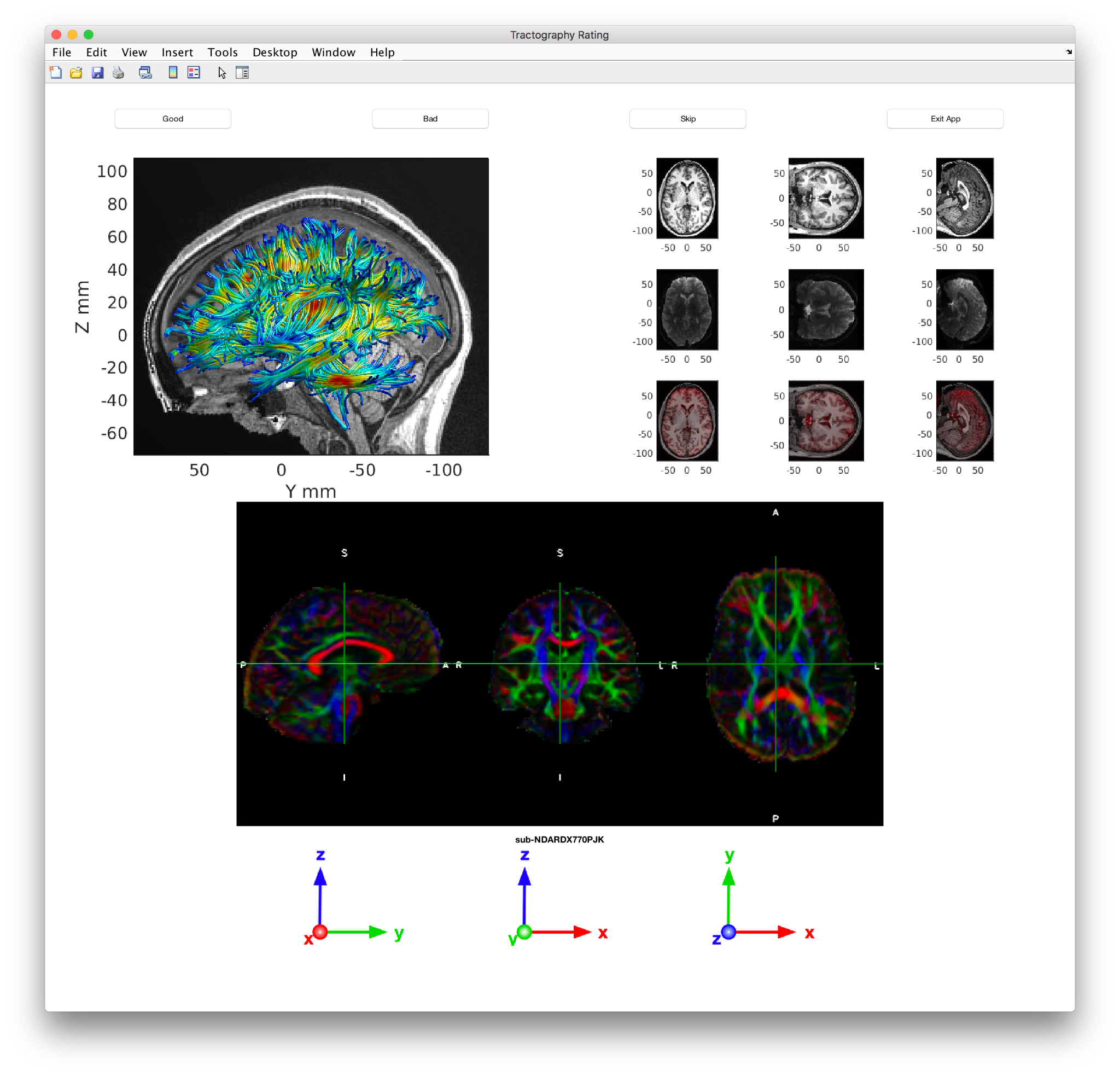

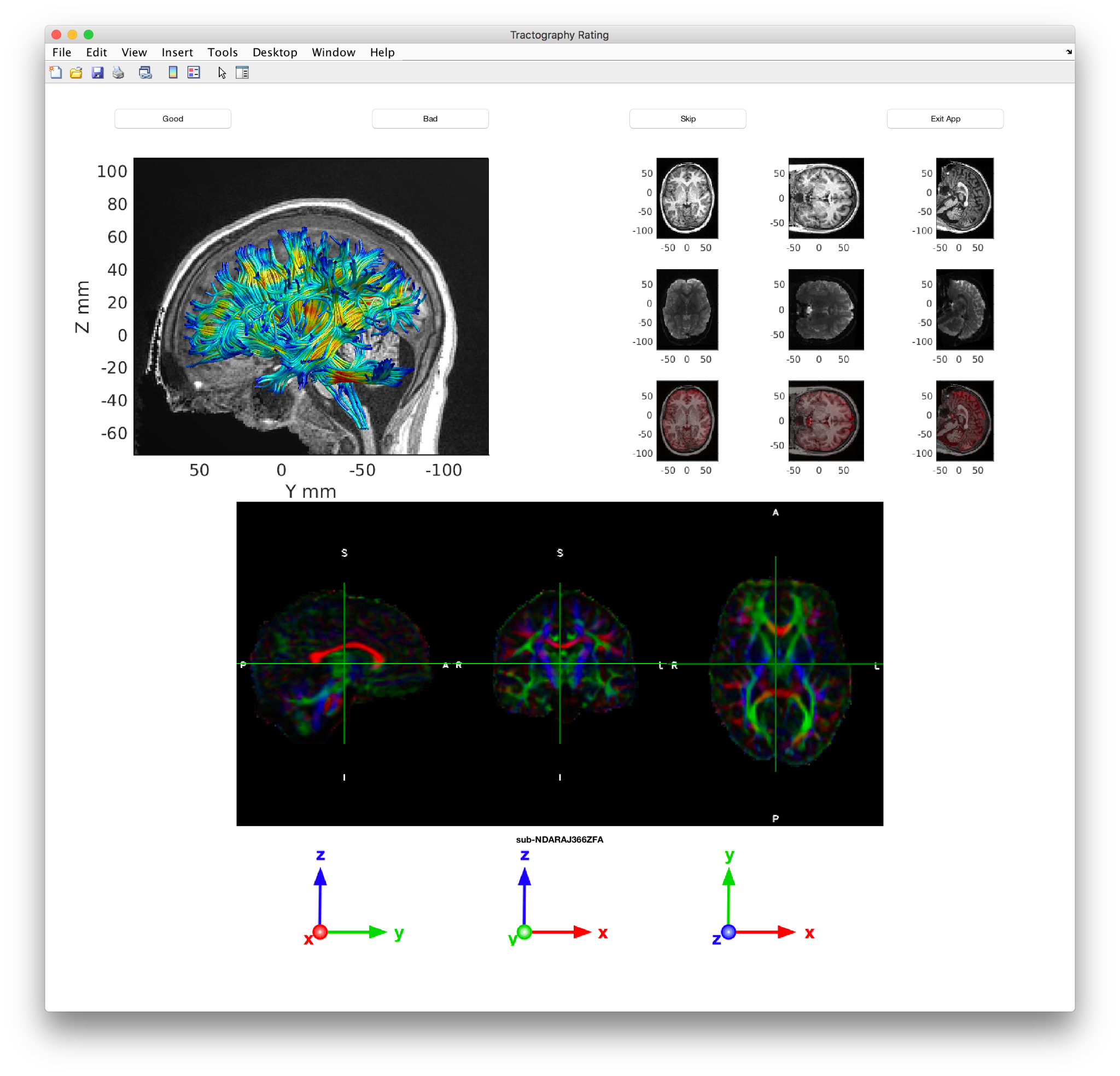


**Figure S2:** Two examples of visual quality control inspection that pass the exclusion criteria (“good”).

**
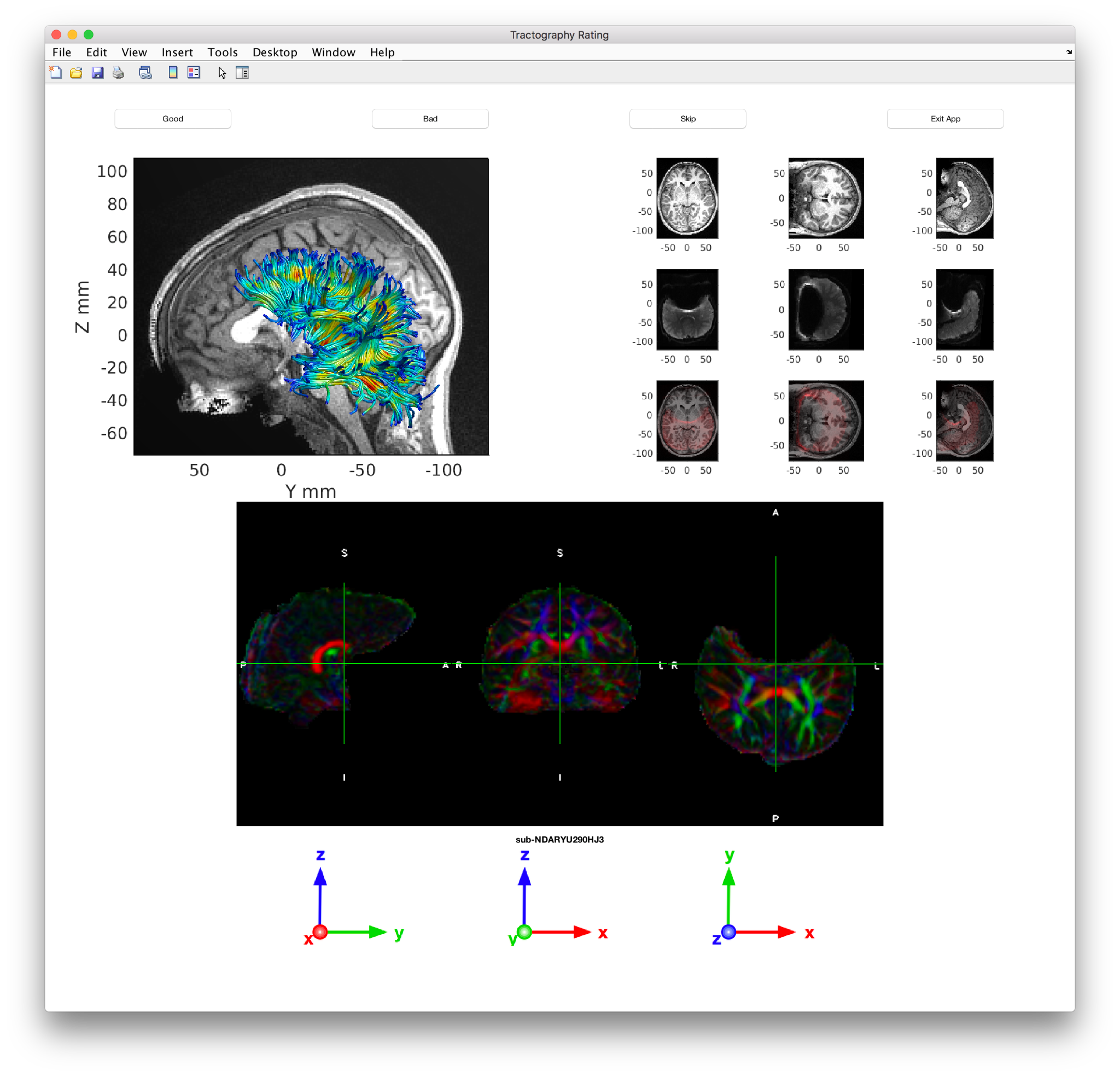

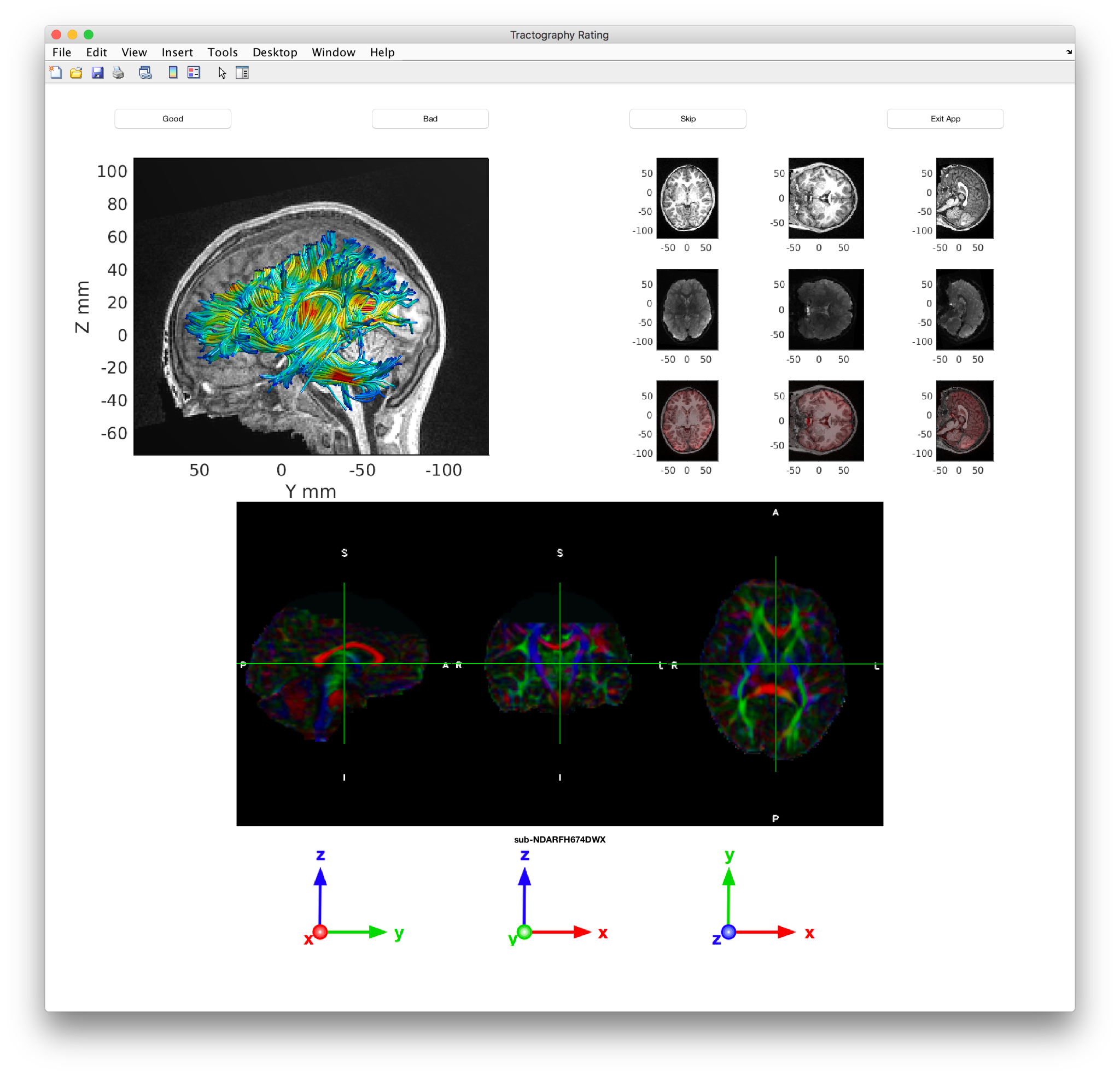
**

**Figure S3:** Two examples of visual quality control inspection that do not pass the exclusion criteria (“bad”). Left: misalignment of DTI and T1-weighted scan and gross artefacts in DTI scan. Right: incomplete tractography with missing areas in the fronto-parietal areas.

# Groupwise Comparison of Demographics, Clinical Characteristics and Head Motion Measures

**Table S2:** Comparison of ADHD-IN and control group. *t*: Unpaired t-test; *d*: Cohen's d; 𝛘2: Pearson's Chi-squared test with Yates' continuity correction; *V*: Cramer's V.

|  | **ADHD-IN**  **(n = 339)** | **Controls**  **(n = 121)** | **Statistical Test** | ***p*** | **Effect Size** |
| --- | --- | --- | --- | --- | --- |
| Age, Years, Mean (SD) | 12.01 (3.34) | 11.14 (3.55) | *t* = 2.40 | 0.017 * | *d* = 0.25 |
| Sex, Female, n (%) | 119 (35.1%) | 56 (46.28%) | 𝛘2 = 4.26 | 0.039 * | *V* = 0.10 |
| IQ, Mean (SD) | 98.21 (15.55) | 109.01 (14.37) | *t* = -6.69 | < 0.001 *** | *d* = -0.71 |
| SWAN, Raw Score, Mean (SD) |  |  |  |  |  |
| Inattention | 1.14 (0.89) | -0.42 (1.14) | *t* = 15.21 | < 0.001 *** | *d* = 1.63 |
| Hyperactivity | 0.14 (0.86) | -0.57 (1.10) | *t* = 7.15 | < 0.001 *** | *d* = 0.77 |
| Total | 0.64 (0.69) | -0.50 (1.02) | *t* = 13.42 | < 0.001 *** | *d* = 1.44 |
| Head Motion, mm, Mean (SD)^a^ |  |  |  |  |  |
| Relative displacement | 0.44 (0.36) | 0.50 (0.38) | *t* = -2.42 | 0.016 * | *d* = -0.25 |
| Absolute displacement | 1.16 (1.22) | 1.17 (1.31) | *t* = -0.12 | 0.907 | *d* = -0.01 |
| ^a^Statistical test calculated on log-transformed values | | | | | |

**Table S3:** Comparison of ADHD-C and control group. *t*: Unpaired t-test; *d*: Cohen's d; 𝛘2: Pearson's Chi-squared test with Yates' continuity correction; *V*: Cramer's V.

|  | **ADHD-C**  **(n = 279)** | **Controls**  **(n = 121)** | **Statistical Test** | ***p*** | **Effect Size** |
| --- | --- | --- | --- | --- | --- |
| Age, Years, Mean (SD) | 10.38 (3.02) | 11.14 (3.55) | *t* = -2.21 | 0.027 * | *d* = -0.24 |
| Sex, Female, n (%) | 61 (21.86%) | 56 (46.28%) | 𝛘2 = 23.15 | < 0.001 *** | *V* = 0.25 |
| IQ, Mean (SD) | 101.15 (15.36) | 109.01 (14.37) | *t* = -4.79 | < 0.001 *** | *d* = -0.52 |
| SWAN, Raw Score, Mean (SD) |  |  |  |  |  |
| Inattention | 1.26 (0.85) | -0.42 (1.14) | *t* = 16.20 | < 0.001 *** | *d* = 1.78 |
| Hyperactivity | 1.13 (0.81) | -0.57 (1.10) | *t* = 16.98 | < 0.001 *** | *d* = 1.87 |
| Total | 1.19 (0.72) | -0.50 (1.02) | *t* = 18.73 | < 0.001 *** | *d* = 2.06 |
| Head Motion, mm, Mean (SD)^a^ |  |  |  |  |  |
| Relative displacement | 0.56 (0.48) | 0.50 (0.38) | *t* = 0.65 | 0.518 | *d* = 0.07 |
| Absolute displacement | 1.53 (1.41) | 1.17 (1.31) | *t* = 2.45 | 0.015 * | *d* = 0.26 |
| ^a^Statistical test calculated on log-transformed values | | | | | |

**Table S4:** Comparison of ADHD-C and ADHD-IN group. *t*: Unpaired t-test; *d*: Cohen's d; 𝛘2: Pearson's Chi-squared test with Yates' continuity correction; *V*: Cramer's V.

|  | **ADHD-C**  **(n = 279)** | **ADHD-IN**  **(n = 339)** | **Statistical Test** | **p** | **Effect Size** |
| --- | --- | --- | --- | --- | --- |
| Age, Years, Mean (SD) | 10.38 (3.02) | 12.01 (3.34) | *t* = 6.31 | < 0.001 *** | *d* = 0.51 |
| Sex, Female, n (%) | 61 (21.86%) | 119 (35.10%) | 𝛘2 = 12.36 | < 0.001 *** | *V* = 0.15 |
| IQ, Mean (SD) | 101.15 (15.36) | 98.21 (15.55) | *t* = -2.35 | 0.019 * | *d* = -0.19 |
| Comorbidities, n (%) |  |  |  |  |  |
| Specific learning disorder | 79 (19.75%) | 88 (19.13%) | 𝛘2 = 0.34 | 0.560 | *V* = 0.03 |
| Autism spectrum disorder | 49 (12.25%) | 58 (12.61%) | 𝛘2 < 0.01 | 0.957 | *V* < 0.01 |
| Oppositional defiant disorder | 80 (20.00%) | 28 (6.09%) | 𝛘2 = 42.96 | < 0.001 *** | *V* = 0.27 |
| Conduct disorder | 3 (0.75%) | 5 (1.09%) | 𝛘2 < 0.01 | 0.939 | *V* = 0.02 |
| Anxiety disorder | 109 (27.25%) | 126 (27.39%) | 𝛘2 = 0.18 | 0.673 | *V* = 0.02 |
| Major depressive disorder | 17 (4.25%) | 37 (8.04%) | 𝛘2 = 3.84 | 0.050 * | *V* = 0.08 |
| Other mental disorder | 66 (16.50%) | 111 (24.13%) | 𝛘2 = 5.65 | 0.017 * | *V* = 0.10 |
| SWAN, Raw Score, Mean (SD) |  |  |  |  |  |
| Inattention | 1.26 (0.85) | 1.14 (0.89) | *t* = -1.65 | 0.099 | *d* = -0.13 |
| Hyperactivity | 1.13 (0.81) | 0.14 (0.86) | *t* = -14.46 | < 0.001 *** | *d* = -1.17 |
| Total | 1.19 (0.72) | 0.64 (0.69) | *t* = -9.65 | < 0.001 *** | *d* = -0.78 |
| Head Motion, mm, Mean (SD)a |  |  |  |  |  |
| Relative displacement | 0.56 (0.48) | 0.44 (0.36) | *t* = 3.62 | < 0.001 *** | *d* = 0.29 |
| Absolute displacement | 1.53 (1.41) | 1.16 (1.22) | *t* = 3.47 | < 0.001 * | *d* = 0.28 |
| ^a^Statistical test calculated on log-transformed values | | | | | |

# Distribution of SWAN Scores


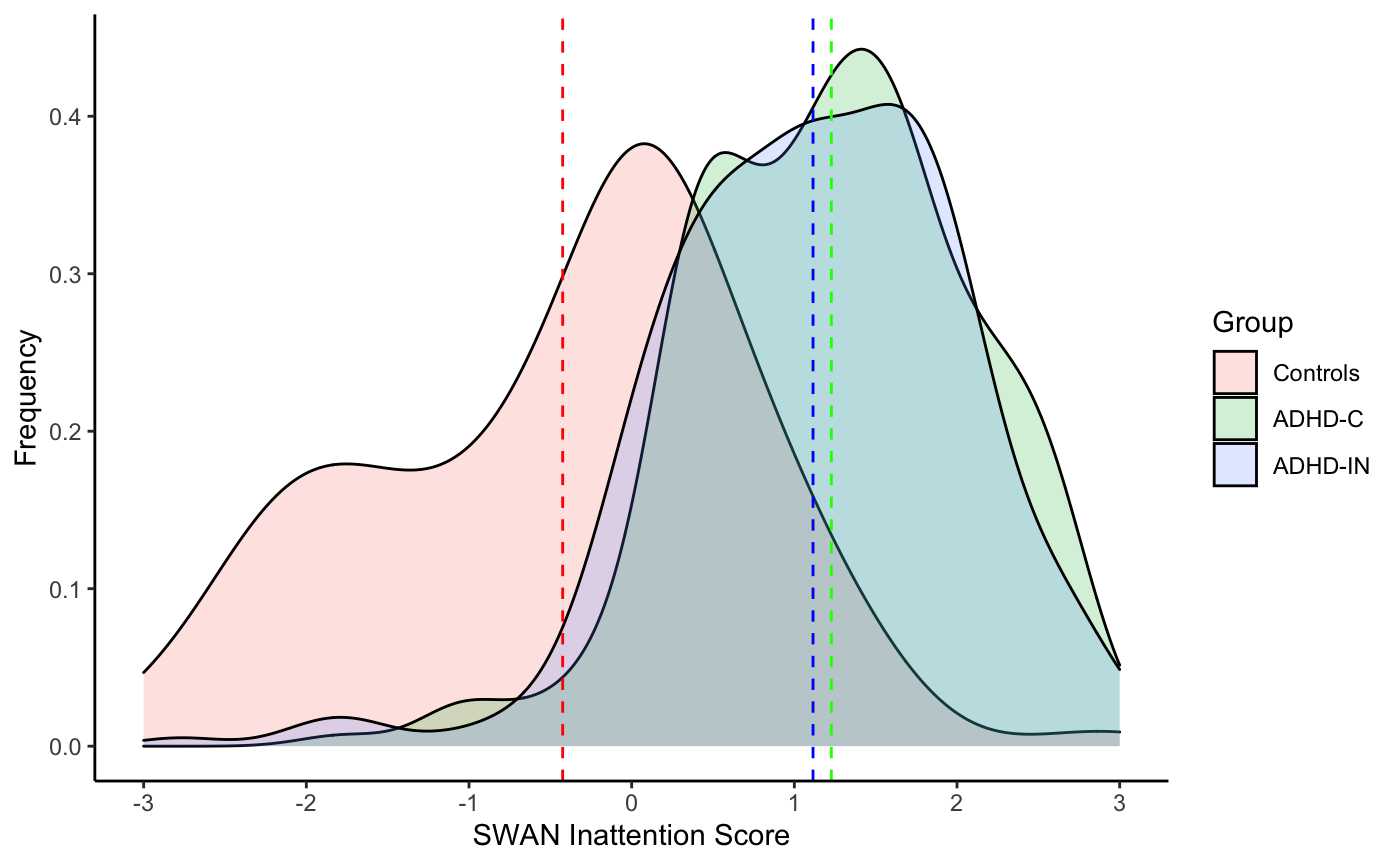


**Figure S2:** Distribution of SWAN Inattention sub-score by study group. Dashed line: group mean; x-axis: -3: very low; -2: low; -1: slightly lower; 0: average; 1: slightly higher; 2: high; 3: very high.


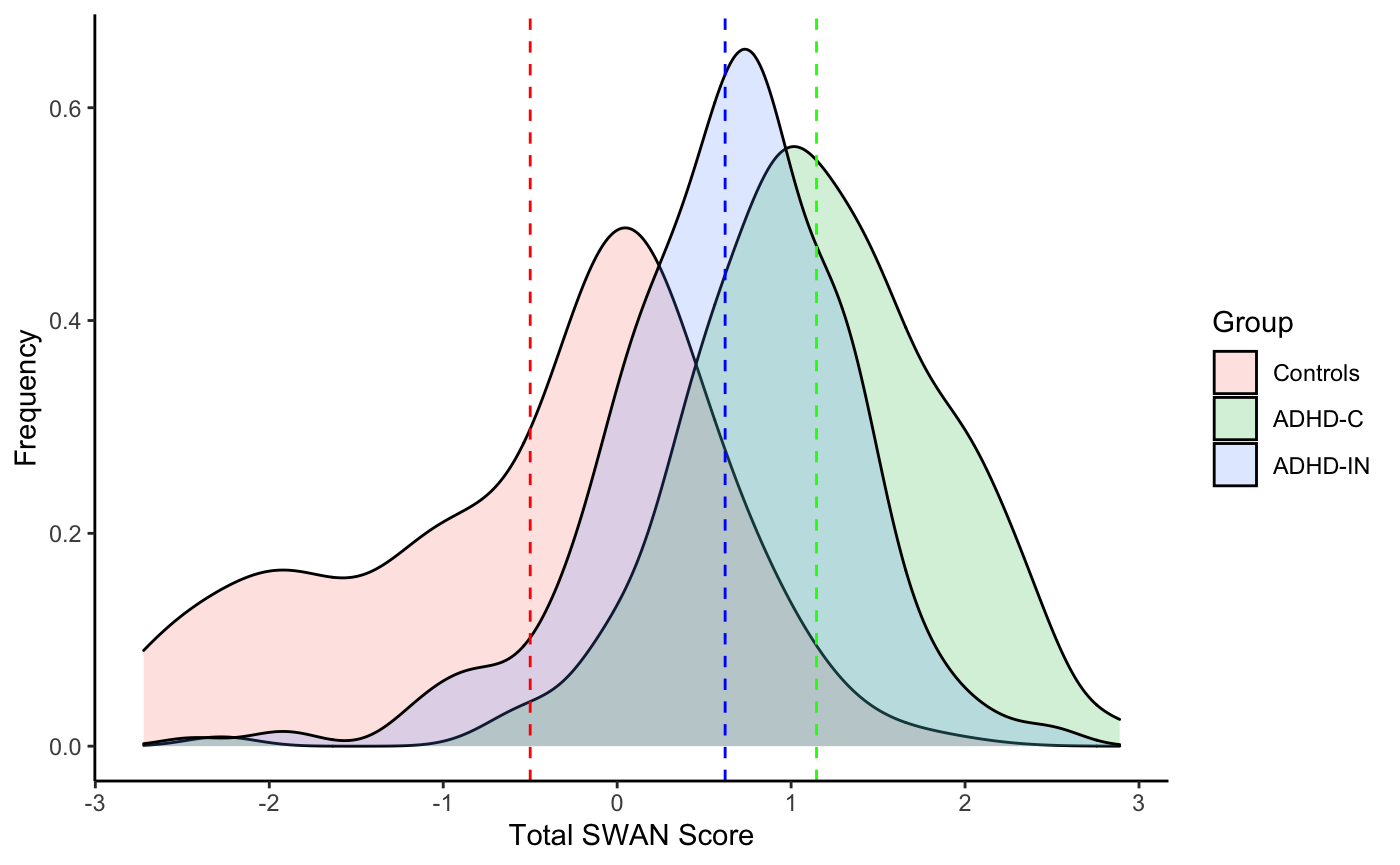


**Figure S3:** Distribution of Total SWAN Score by study group. Dashed line: group mean; x-axis: -3: very low; -2: low; -1: slightly lower; 0: average; 1: slightly higher; 2: high; 3: very high.

# MRI Scanning Parameters

MRI data were recorded at three acquisition sites (Staten Island, Rutgers University Brain Imaging Center, and CitiGroup Cornell Brain Imaging Center) with different scanning parameters and equipment. Full scanning protocols are available at <http://fcon_1000.projects.nitrc.org/indi/cmi_healthy_brain_network/> and in [26].

## Staten Island (SI) Scanning Site

This scanning site was a mobile trailer in the Staten Island area. The mobile trailer was equipped with a 1.5 T Siemens Avanto system with 45 mT/m gradients, 32 RF receive channels, and the Siemens 32-channel head coil and acquired data using the University of Minnesota Center for Magnetic Resonance Research (CMRR) simultaneous multi-slice echo-planar imaging sequence. Diffusion kurtosis imaging (DKI) scan parameters were set to 72 slices, with a resolution of 2 × 2 × 2 mm, TR = 3110 ms, TE = 76.2 ms, flip angle of 90 degrees, threefold multiband acceleration, in 64 diffusion directions and b-values of 0, 1000, and 2000 s/mm^2^. T1-weighted imaging scans comprised 176 slices at a resolution of 1 × 1 × 1 mm, TR = 2730 ms, TE = 1.64 ms, TI = 1000 ms and a flip angle of 7 degrees. The total scanning duration was approximately 62 minutes with the DTI scanning unit starting at minute 21 and lasting about 10 minutes.

## Rutgers University Brain Imaging Center (RUBIC) Scanning Site

At the Rutgers University Brain Imaging Center, data were collected using a Siemens 3T Tim Trio MRI scanner with a Siemens 32-channel head coil and the CMRR simultaneous multi-slice echo-planar imaging sequence for acquisition. At this site, DKI was acquired with 72 slices, at a resolution of 1.8 × 1.8 × 1.8 mm, TR = 3320 ms, TE = 100.2 ms, a flip angle of 90 degrees, threefold multiband acceleration, in 64 diffusion directions and b-values of 0, 1000, and 2000 s/mm^2^. The scan parameters for T1-weighted imaging at RUBIC were 224 slices at a resolution of 0.8 × 0.8 × 0.8 mm, TR = 2500 ms, TE = 3.15 ms, TI = 1060 ms, and flip angle of 8 degrees. The total scanning duration differed for scans from release 1 to release 4, and scans from release 5 onwards. For the first releases, scanning lasted 66 minutes; the DKI scanning unit started at minute 32 and took approximately 9.5 minutes. For the later releases, total scanning time was 54 minutes with the DKI scan starting at minute 46 and lasting about 8.5 minutes.

## CitiGroup Cornell Brain Imaging Center (CBIC) Scanning Sites

The CitiGroup Cornell Brain Imaging Center site used a 3T Prisma scanner with a Siemens 32-channel head coil and the CMRR simultaneous multi-slice echo-planar imaging sequence for acquisition was applied. DKI was acquired with the following parameters: 81 slices, a resolution of 1.8 × 1.8 × 1.8 mm, TR = 3320 ms, TE = 100.2 ms, a flip angle of 90 degrees, threefold multiband acceleration, 64 diffusion directions, and b-values of 0, 1000, and 2000 s/mm^2^. T1-weighted imaging was acquired with identical scan parameters as at the RUBIC site. Also here, the total scanning duration differed for scans being from release 1 to release 4, and scans from release 5 onwards. For all releases, scanning lasted 75 minutes, with the DKI scanning unit starting at minute 33 for the first four releases and lasting approximately 9.5 minutes, and starting at minute 60 and lasting about 10 minutes for the later releases.

# MRI Preprocessing and System Specifications

Preprocessing included the following steps: First, DTI scans were denoised with the “MPdenoising” function, which is a 4-dimensional block processing image denoising with the default kernel and noise map estimation algorithm [27]. Next, the denoised images were corrected for Gibbs artefacts with the “unring” function [28]. Gibbs artefacts are oscillations close to high-contrast regions in the image. Susceptibility-induced off-resonance field distortion correction, which corrects for artefacts predominantly at tissue boundaries [29], was omitted at this stage in favor of including a larger sample for whom echo-planar image (EPI) scans with reversed phase-encode blips were unavailable. For a pre-analysis of the effect of omitting susceptibility-induced off-resonance field distortion correction on the estimation of tract-wise mean FA in the selected structures, see Supplement G. For the following steps, FMRIB Software Library (FSL) version 6.0.4 was used [30]. The FSL Brain Extraction Tool (BET) was applied to extract brain tissue from nonbrain tissue in the whole head image using an FA threshold of 0.1, yielding a binary brain mask [31]. Eddy current-induced distortions, which are a common artefact of diffusion images, were removed from the data using the eddy_cuda FSL tool. This tool also corrects artefacts of participants’ in-scanner head movements [32–34]. Eddy current-induced distortions and head movement correction was computed in 8 iterations using decreasing full-width-half-max parameters for smoothing in each iteration. Outlier slices were detected and replaced in slice-wise outliers and multiband group outliers. Slice-wise outliers are signal dropouts that occur within a single slice, whereas multiband group outliers are signal dropouts across all slices acquired simultaneously within the group of a multiband acquisition. Correcting for multiband group outliers was particularly important for this study because at all three sites, slices were acquired with a threefold multiband acceleration. In addition to within-volume correction, this eddy version also performs slice-to-volume correction, which takes movements into account that occur within a volume instead of between volumes, modeled by 9 degrees of freedom. The additional movement correction outperforms previous implementations of the eddy tool by further limiting the impact of remaining distortions on subsequently extracted diffusivity measures, which is why this version was chosen over previous ones [32–34]. Nonetheless, it is critical to note that, although we chose the latest version of movement correction including both slice-to-volume and within-volume artefacts, residual head movement effects remained because the true movement can only be estimated, and the ground truth remains unknown. We support the view that these residual artefacts are crucial in influencing subsequently derived measures of white matter integrity [35–37]. Consequently, we used the estimated in-scanner head motion from motion correction in the subsequent causal mediation analysis. Preprocessing continued with outlier detection and robust estimation of MRI parameters (function “irlls”) using iterative reweighted linear least squares [38]. Tensor fitting and extraction of diffusivity measures were applied with weighted linear least squares estimation as implemented in the function “dki_fit” [39]. DKI parameters were extracted with the function “dki_parameters” [40]. Lastly, we used the function “wmti_parameters” to calculate white matter tract integrity metrics [41].

## Tractography

We used Automating fiber-tract quantification (AFQ) (v.1.1) [42], which is a deterministic streamline tracking algorithm (STT) [43, 44], to extract diffusion properties as indicators for white matter integrity. We have chosen deterministic tractography over voxel-based analyses and tract-based spatial statistics (TBSS) to overcome insufficient precision in co-registration due to large natural variability in fiber morphology [42, 45, 46]. Tractography allows the shape of the entire tract to be tracked regardless of its morphology and is therefore most suitable for studying populations with potentially strong deviations in tract size and shape [42, 46]. To maintain comparability between participants, we chose to analyze tract properties on clipped regions of interest, which are areas defined between two anatomical waypoints specific to the tract [42, 47]. We focused on the diffusivity measure of FA because it has been used in the majority of previous studies on children with ADHD [35, 48, 49]. Furthermore, FA is commonly interpreted as an indicator of myelination, because axonal cell membranes and surrounding myelin sheath force Brownian water diffusion to be anisotropic in the direction of the fiber tract [50]. With exceptions of fiber crossings and variations in axonal density and thickness [51], interparticipant deviations in FA are commonly indicative of deviation in the degree of myelination [52]. Thus, we choose FA to reflect white matter integrity in the tracts of interest. We used the same diffusivity measure to investigate whether the discrepancies in past ADHD studies are due to an underestimation of the impact of in-scanner head motion.

## System Specifications

Preprocessing of MRI Data was performed on a system with the following specifications:

**Graphics Card:** NVIDIA Tesla P4

**Graphics Processor Name:** GP104

**Graphics Processor Variant:** GP104-895-A1

**Architecture:** Pascal

**Onboard Memory Size:** 8 GB

**Onboard Memory Type:** GDDR5

**Accelerator:** 1x nVidia Tesla P4

**NVRM version:** NVIDIA UNIX x86_64 Kernel Module

**GCC version:** gcc version 7.5.0

**Cuda Version:** 9.1.85

**Hypervisor CPU:** Intel Xeon Gold 6126

**Number of CPUs:** 8

**GHz:** 2.60

**Architecture:** x86_64

**System Memory:** 62 GB DIMM RAM

**Operating system:** Ubuntu 18.04.5 LTS (Bionic Beaver)

# Pre-Analysis of Necessity of Susceptibility-Induced Off-Resonance Field Distortion Correction

The diffusion parameter estimation with Gibbs and noise removal (DESIGNER) pipeline [53] recommends susceptibility-induced off-resonance field distortion correction, for which two echo-planar image (EPI) scans with reversed phase-encode blips are needed. In our sample, not all participants completed the full scanning procedure. Thus, we tested the benefit of omitting this correction step and including more participants in our sample who did not complete field map acquisitions. Furthermore, susceptibility-induced off-resonance field distortions are most prominent at the interface of tissue boundaries: between air, bone, and brain, in the direction of the phase-encode blips [29]. The EPI scans in this sample were acquired with A>>P and P>>A phase-encode blips. Therefore, the areas that are expected to be most affected by the distortions are around the tissue boundaries of the frontal lobe and the occipital lobe. As we are interested in the deeper located white matter tracts and in further processing steps, we clipped the measurements of the tracts to predefined regions of interest, thereby excluding parts of the tract that are in the periphery of the brain. We hypothesize that susceptibility-induced off-resonance field distortion correction will not have a major impact on the measures of FA derived from these regions.

To assess the necessity of susceptibility-induced off-resonance field distortion correction for the quantification of deeper structures, we ran the entire DESIGNER preprocessing pipeline [53] with and without this correction on a subsample of our data and compared the FA measures. We ran the preprocessing pipeline once including the FSL “topup” command [54] and fed the output into later eddy-current correction, and we ran the exact same pipeline once without the “topup” command. We confirmed that the sample we chose for this test was representative by subsampling and matching for age and in-scanner head motion. Participants for this subsample were also drawn in proportion to the number of participants at each scanning site. We confirmed this hypothesis by inspecting the tract profile with and without susceptibility-induced off-resonance field distortion correction of the six tracts of interest averaged across all participants in the subsample. The following Figures S8–S13 show a comparison of the FA tract profiles derived from those tracts.


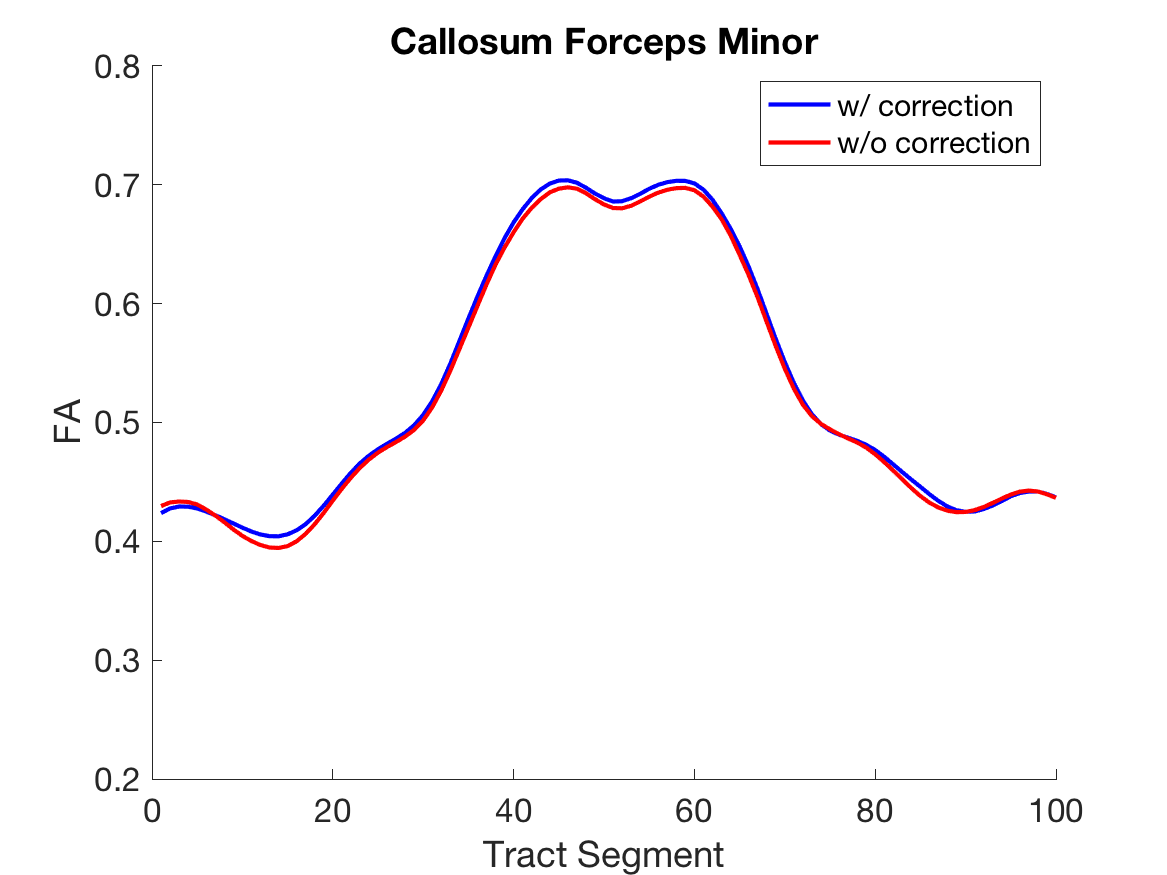

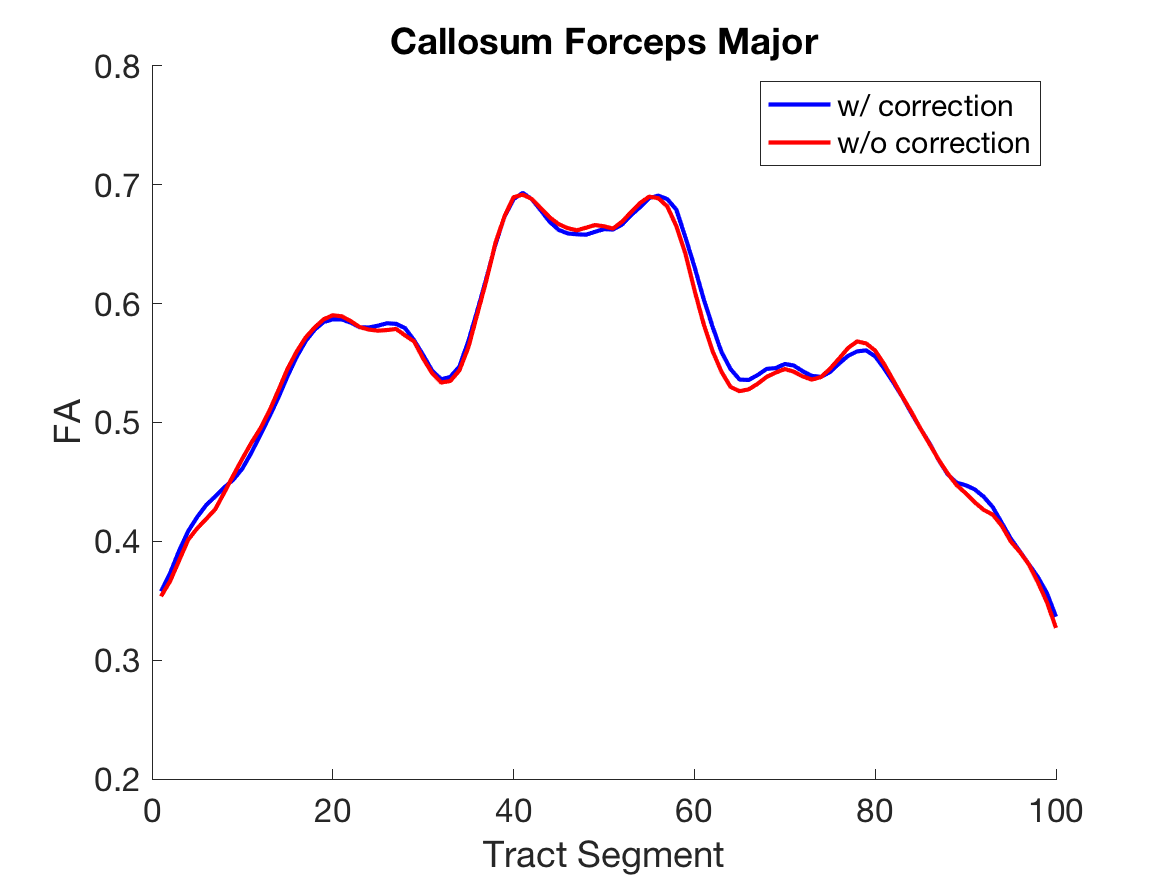

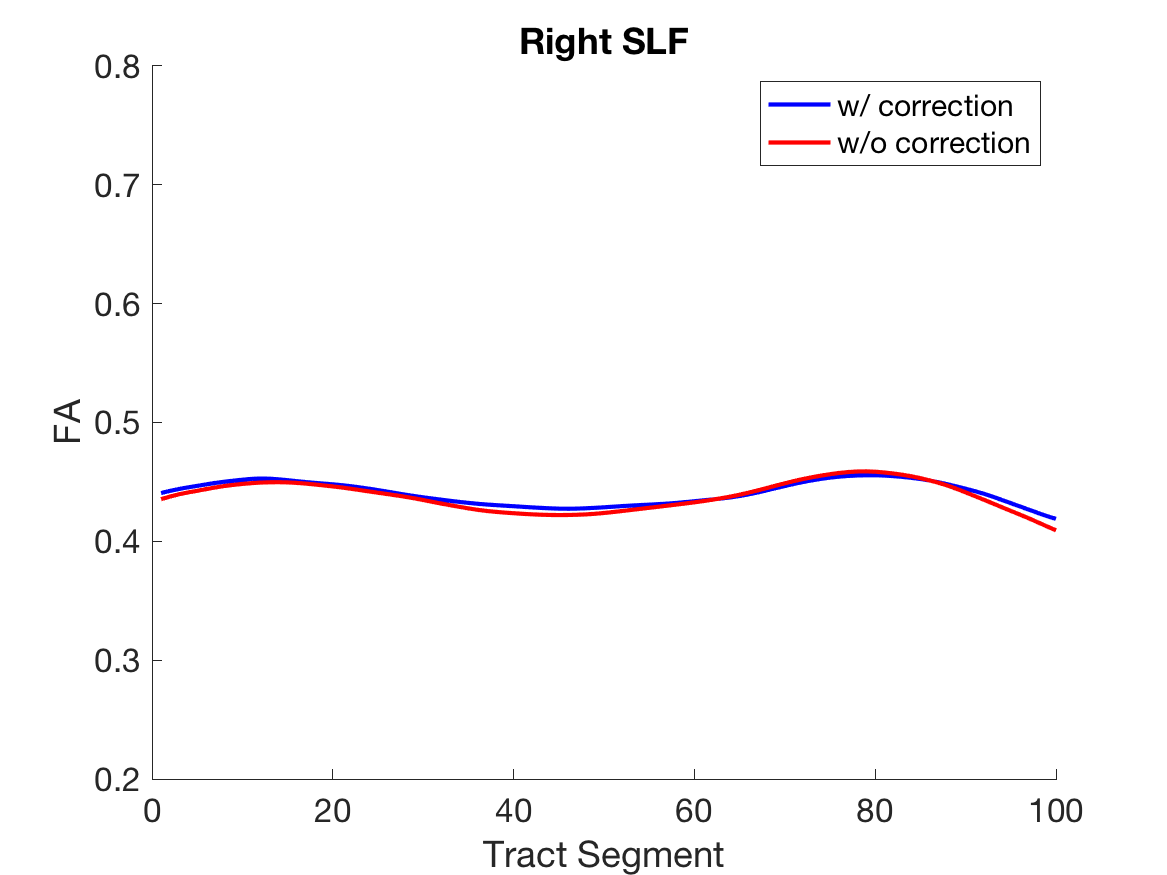

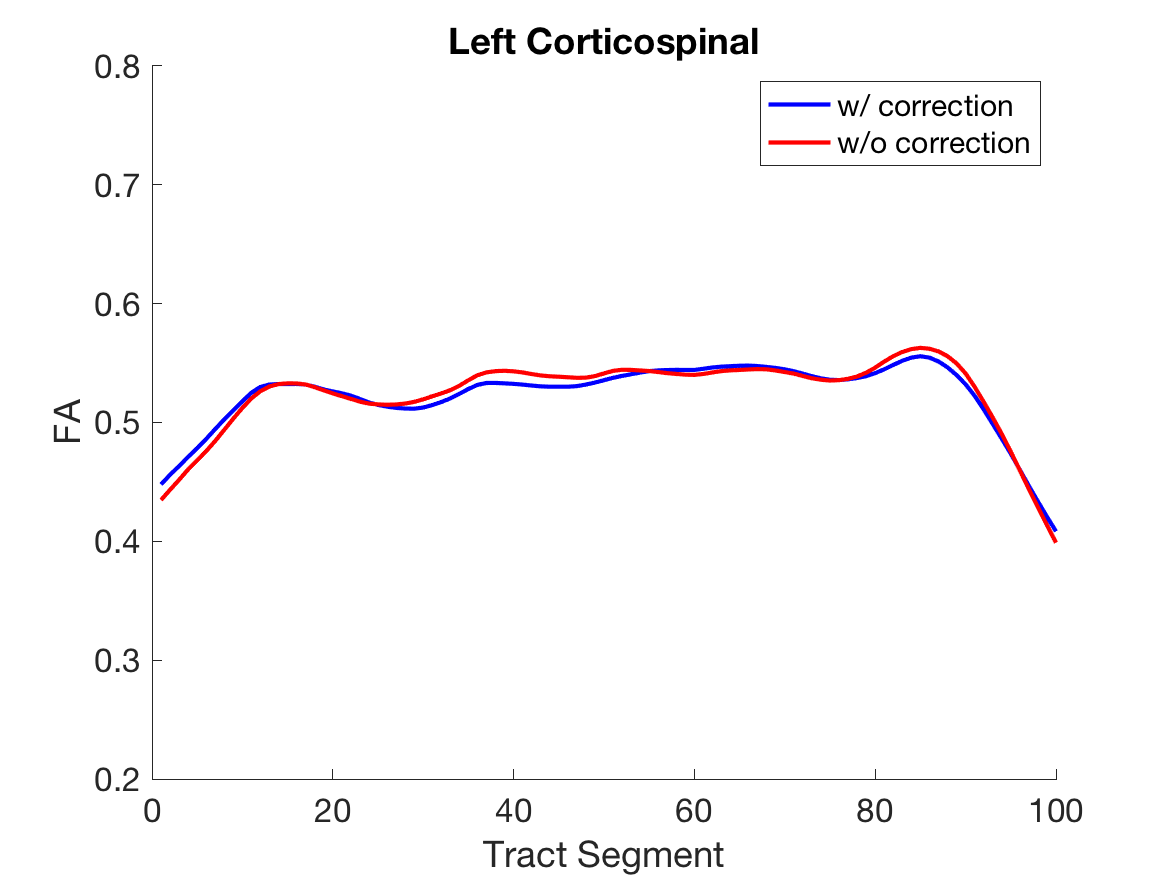

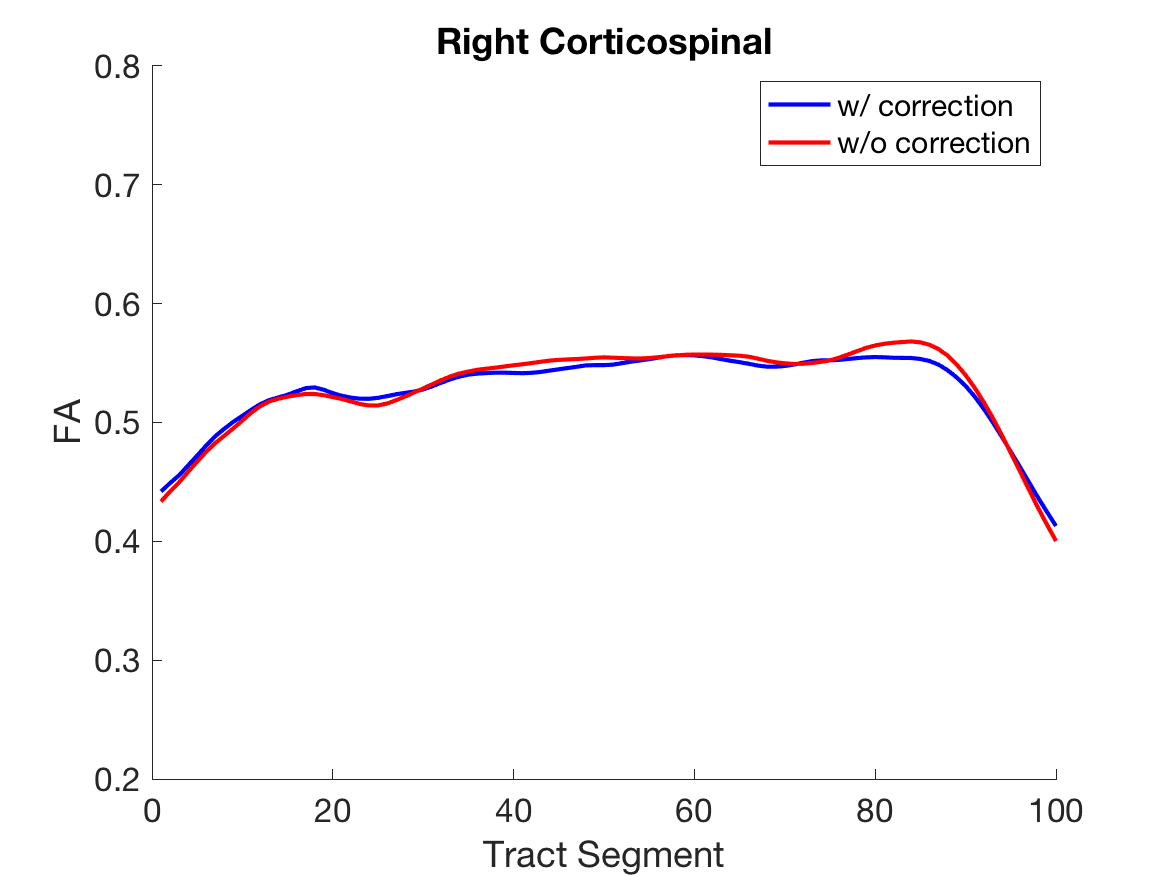

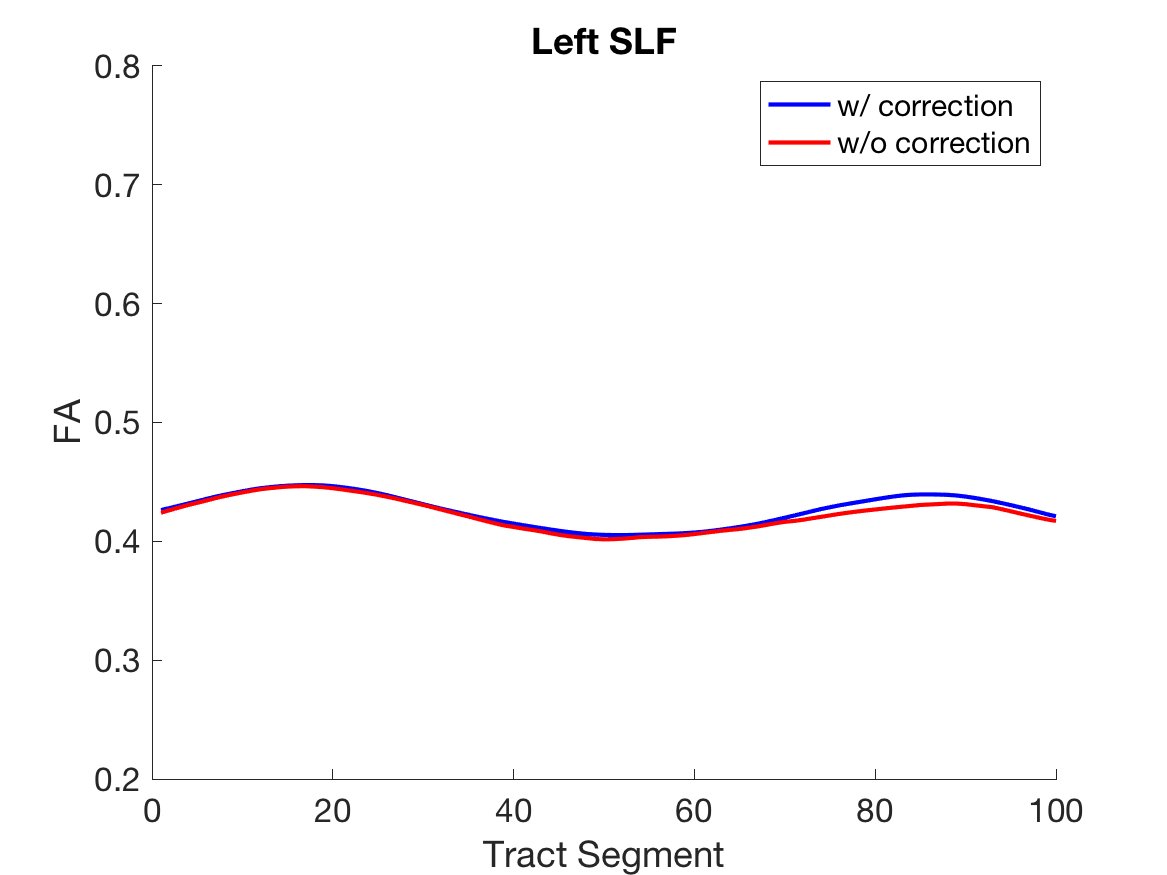


**Figure S4:** Mean tract profile of corpus callosum forceps minor and major, left and right corticospinal tract (CST), and left and right superior longitudinal fasciculus (SLF) with (blue) and without (red) susceptibility-induced off-resonance field distortion correction during preprocessing.

From the above graphs we conclude that susceptibility-induced off-resonance field distortion correction does not affect FA for the selected structures at the clipped regions of interest. If susceptibility-induced off-resonance field distortion correction had an outstanding effect, it would have been most obvious for the corpus callosum forceps minor and forceps major at the first and last few tract segments as these are the regions closest to tissue boundaries and are aligned with the acquisition direction (i.e., A >> P and P >> A). However, in those regions we did not observe any clear differences between the result with and without susceptibility-induced off-resonance field distortion correction. Consequently, we decided to increase the sample size in this study for which there were no field map scans available in favor of correcting for susceptibility-induced off-resonance field distortions.

# Nested Model Comparison

In all following formulas, effects are denoted in accordance with the Wilkinson notation [55]. Since in-scanner head motion (continuous) is a variable of main interest, and age (continuous), sex (categorical: male, female), IQ (continuous) and site (categorical: SI, RUBIC, CBIC) are considered necessary confounding factors, we defined the base model as follows:

**Model 1:** Whole-Brain FA ~ Age + Sex + IQ + Site + Motion

## Categorical Analysis

We compared extended models with respect to the base model to identify the benefit of including the ADHD category (categorical, 3 levels) as a fixed effect or an interaction effect between in-scanner head motion and the ADHD category. The ADHD category was either “ADHD-IN”, “ADHD-C” or “No ADHD“ for the control participants, respectively. Consequently, Model 2 and Model 3 were defined as follows:

**Model 2:** Whole-Brain FA ~ Age + Sex + IQ + Site + Motion + ADHD Category

**Model 3:** Whole-Brain FA ~ Age + Sex + IQ + Site + Motion * ADHD Category

A comparison of the models using ANOVA yielded the following results:

**Table S5:** Analysis of variance table for categorical models using the ADHD category.

|  | **Res.Df** | **RSS** | **Df** | **Sum of Sq** | **F** | **p** |
| --- | --- | --- | --- | --- | --- | --- |
| **Model 1** | 729 | 0.265 |  |  |  |  |
| **Model 2** | 727 | 0.265 | 2 | 0.00059 | 0.81 | 0.446 |
| **Model 3** | 725 | 0.263 | 2 | 0.00180 | 2.49 | 0.084 |

In the categorical analysis, none of the extended models including ADHD category as a fixed effect and an interaction effect of ADHD category and in-scanner head motion outperformed the base model. Therefore, no significant main effects of the ADHD category in the categorical analysis were neither expected, nor obtained (see Supplement I Table S7-S13). Consequently, a categorical approach was not expected to be suitable for causal mediation analysis.

## Dimensional Analysis

In the dimensional analysis we performed the same comparison as in the categorical analysis, but using the SWAN-Hyperactivity sub-score (SWAN-HY; continuous) instead of the ADHD category. Thus, we compared the following extended models with respect to the base model to identify the benefit of including SWAN-HY as a fixed effect and an interaction effect between in-scanner head motion and SWAN-HY.

**Model 2:** Whole-Brain FA ~ Age + Sex + IQ + Site + Motion + SWAN-HY

**Model 3:** Whole-Brain FA ~ Age + Sex + IQ + Site + Motion * SWAN-HY

A comparison of the models using ANOVA yielded the following results:

**Table S6:** Analysis of variance table for dimensional analysis using the SWAN-Hyperactivity sub-score.

|  | **Res.Df** | **RSS** | **Df** | **Sum of Sq** | **F** | **p** |
| --- | --- | --- | --- | --- | --- | --- |
| **Model 1** | 721 | 0.263 |  |  |  |  |
| **Model 2** | 720 | 0.261 | 1 | 0.00137 | 3.78 | 0.052 |
| **Model 3** | 719 | 0.261 | 1 | 0.00072 | 1.99 | 0.159 |

Consequently, we concluded to reject a model with an interaction effect between in-scanner head motion and SWAN-HY (Model 3). Although Model 2 turned out only marginally better in explaining the variance in the data than the base model, we decided to include SWAN-HY as a main effect since it is a variable of interest and turned out significant in tract-wise analysis (see Supplement J), rather than on whole-brain FA.

Therefore, we concluded to proceed with the following final model design:

**Model Dim:** FA ~ Age + Sex + IQ + Site + Motion + SWAN-HY

For reasons of completeness, as for the categorical model (Model Cat), we provided the results for the dimensional model (Model Dim) for whole-brain and tract-wise mean FA on all tracts of interest in Supplement J Table S14–S20.

# Categorical Analysis of Full Model

**Model Cat:** FA ~ Age + Sex + IQ + Site + Motion + ADHD Category

## Whole-Brain

**Table S7:** Categorical generalized linear mixed effects model (Model Cat) for whole-brain FA.

| **Variable** | **β** | **SE** | **t** | **p** | **95% CI** | **f^2^** |
| --- | --- | --- | --- | --- | --- | --- |
| Age | 0.0016 | 0.0002 | 7.24 | < 0.001 *** | [0.00120, 0.00209] | 0.0720 |
| Sex | 0.0010 | 0.0015 | 0.67 | 0.507 | [-0.00200, 0.00405] | 0.0006 |
| IQ | <0.0001 | <0.0001 | -0.02 | 0.981 | [-0.00009, 0.0001] | < 0.0001 |
| Site RUBIC | 0.0395 | 0.0016 | 24.79 | < 0.001 *** | [0.03633, 0.04257] | 1.8971 |
| Site SI | 0.0838 | 0.0024 | 35.21 | < 0.001 *** | [0.07911, 0.08846] | 1.8971 |
| Motion | -0.0066 | 0.0011 | -5.86 | < 0.001 *** | [-0.00877, -0.00437] | 0.0472 |
| ADHD-IN | -0.0004 | 0.0022 | -0.20 | 0.840 | [-0.00469, 0.00381] | 0.0022 |
| ADHD-C | -0.0022 | 0.0022 | -1.01 | 0.312 | [-0.00651, 0.00208] | 0.0022 |

SE: standard error; *f^2^*: Cohen’s f-squared.

## Corpus Callosum Forceps Minor

**Table S8:** Categorical generalized linear mixed effects model (Model Cat) for corpus callosum forceps minor.

| **Variable** | **β** | **SE** | **t** | **p** | **95% CI** | **f^2^** |
| --- | --- | --- | --- | --- | --- | --- |
| Age | 0.0009 | 0.0004 | 2.01 | 0.045 * | [0.00002, 0.00173] | 0.0056 |
| Sex | 0.0003 | 0.0030 | 0.10 | 0.921 | [-0.00553, 0.00612] | < 0.0001 |
| IQ | 0.0002 | 0.0001 | 1.82 | 0.069 | [-0.00001, 0.00034] | 0.0046 |
| Site RUBIC | 0.0558 | 0.0031 | 18.29 | < 0.001 *** | [0.04981, 0.06179] | 0.5952 |
| Site SI | 0.0751 | 0.0046 | 16.43 | < 0.001 *** | [0.06613, 0.08408] | 0.5952 |
| Motion | -0.0141 | 0.0021 | -6.58 | < 0.001 *** | [-0.01834, -0.00991] | 0.0597 |
| ADHD-IN | -0.0031 | 0.0042 | -0.74 | 0.457 | [-0.01127, 0.00508] | 0.0049 |
| ADHD-C | -0.0073 | 0.0042 | -1.73 | 0.084 | [-0.01555, 0.00099] | 0.0049 |

SE: standard error; *f^2^*: Cohen’s f-squared.

## Corpus Callosum Forceps Major

**Table S9:** Categorical generalized linear mixed effects model (Model Cat) for corpus callosum forceps major.

| **Variable** | **β** | **SE** | **t** | **p** | **95% CI** | **f^2^** |
| --- | --- | --- | --- | --- | --- | --- |
| Age | 0.0011 | 0.0007 | 1.59 | 0.112 | [-0.00027, 0.00254] | 0.0038 |
| Sex | -0.0068 | 0.0048 | -1.41 | 0.159 | [-0.01631, 0.00268] | 0.0030 |
| IQ | < 0.0001 | 0.0001 | 0.11 | 0.912 | [-0.00027, 0.00031] | < 0.0001 |
| Site RUBIC | 0.0353 | 0.0051 | 6.97 | < 0.001 *** | [0.02532, 0.04520] | 0.1050 |
| Site SI | 0.0528 | 0.0075 | 7.03 | < 0.001 *** | [0.03808, 0.06760] | 0.1050 |
| Motion | -0.0073 | 0.0359 | -2.04 | 0.042 * | [-0.01440, -0.00028] | 0.0063 |
| ADHD-IN | 0.0021 | 0.0068 | 0.31 | 0.755 | [-0.01127, 0.01552] | 0.0002 |
| ADHD-C | 0.0020 | 0.0069 | 0.29 | 0.769 | [-0.01153, 0.01559] | 0.0002 |

SE: standard error; *f^2^*: Cohen’s f-squared.

## Left Corticospinal Tract

**Table S10:** Categorical generalized linear mixed effects model (Model Cat) for left corticospinal tract.

| **Variable** | **β** | **SE** | **t** | **p** | **95% CI** | **f^2^** |
| --- | --- | --- | --- | --- | --- | --- |
| Age | 0.0012 | 0.0005 | 2.60 | 0.010 ** | [0.00030, 0.00214] | 0.0096 |
| Sex | 0.0038 | 0.0032 | 1.18 | 0.237 | [-0.00247, 0.00999] | 0.0020 |
| IQ | -0.0001 | 0.0001 | -1.13 | 0.258 | [-0.00030, 0.00008] | 0.0018 |
| Site RUBIC | 0.0756 | 0.0033 | 23.02 | < 0.001 *** | [0.06914, 0.08203] | 1.5352 |
| Site SI | 0.1499 | 0.0049 | 30.74 | < 0.001 *** | [0.14028, 0.15943] | 1.5352 |
| Motion | -0.0039 | 0.0023 | -1.79 | 0.090 | [-0.00847, 0.00061] | 0.0041 |
| ADHD-IN | 0.0003 | 0.0044 | 0.06 | 0.951 | [-0.00845, 0.00900] | 0.0016 |
| ADHD-C | -0.0032 | 0.0045 | -0.70 | 0.484 | [-0.01198, 0.00568] | 0.0016 |

SE: standard error; *f^2^*: Cohen’s f-squared.

## Right Corticospinal Tract

**Table S11:** Categorical generalized linear mixed effects model (Model Cat) for right corticospinal tract.

| **Variable** | **β** | **SE** | **t** | **p** | **95% CI** | **f^2^** |
| --- | --- | --- | --- | --- | --- | --- |
| Age | 0.0010 | 0.0005 | 2.18 | 0.030 * | [0.00010, 0.00194] | 0.0067 |
| Sex | -0.0002 | 0.0031 | -0.07 | 0.942 | [-0.00640, 0.00594] | < 0.0001 |
| IQ | -0.0001 | 0.0001 | -1.48 | 0.140 | [-0.00034, 0.00005] | 0.0031 |
| Site RUBIC | 0.0917 | 0.0033 | 28.12 | < 0.001 *** | [0.08527, 0.09807] | 1.9733 |
| Site SI | 0.1641 | 0.0049 | 33.80 | < 0.001 *** | [0.15454, 0.17360] | 1.9733 |
| Motion | -0.0049 | 0.0023 | -2.12 | 0.035 * | [-0.00941, -0.00036] | 0.0063 |
| ADHD-IN | -0.0041 | 0.0044 | -0.91 | 0.361 | [-0.01278, 0.00466] | 0.0022 |
| ADHD-C | -0.0056 | 0.0045 | -1.25 | 0.212 | [-0.01439, 0.00320] | 0.0022 |

SE: standard error; *f^2^*: Cohen’s f-squared.

## Left Superior Longitudinal Fasciculus

**Table S12:** Categorical generalized linear mixed effects model (Model Cat) for left superior longitudinal fasciculus.

| **Variable** | **β** | **SE** | **t** | **p** | **95% CI** | **f^2^** |
| --- | --- | --- | --- | --- | --- | --- |
| Age | 0.0024 | 0.0005 | 4.52 | < 0.001 *** | [0.00135, 0.00341] | 0.0286 |
| Sex | 0.0072 | 0.0036 | 2.03 | 0.043 * | [0.00023, 0.01420] | 0.0057 |
| IQ | -0.0002 | 0.0001 | -1.47 | 0.142 | [-0.00037, 0.00005] | 0.0030 |
| Site RUBIC | 0.0190 | 0.0037 | 5.18 | < 0.001 *** | [0.01182, 2.62241] | 0.2379 |
| Site SI | 0.0702 | 0.0055 | 12.88 | < 0.001 *** | [0.05954, 0.08096] | 0.2379 |
| Motion | -0.0080 | 0.0026 | -3.11 | 0.002 ** | [-0.01310, -0.00296] | 0.0135 |
| ADHD-IN | -0.0049 | 0.0050 | -0.99 | 0.322 | [-0.01476, 0.00486] | 0.0017 |
| ADHD-C | -0.0054 | 0.0050 | -1.07 | 0.283 | [-0.01534, 0.00449] | 0.0017 |

SE: standard error; *f^2^*: Cohen’s f-squared.

## Right Superior Longitudinal Fasciculus

**Table S13:** Categorical generalized linear mixed effects model (Model Cat) for right superior longitudinal fasciculus.

| **Variable** | **β** | **SE** | **t** | **p** | **95% CI** | **f^2^** |
| --- | --- | --- | --- | --- | --- | --- |
| Age | 0.0029 | 0.0005 | 5.61 | < 0.001 *** | [0.00186, 0.00387] | 0.0436 |
| Sex | 0.0037 | 0.0035 | 1.07 | 0.286 | [-0.00311, 0.01052] | 0.0016 |
| IQ | < 0.0001 | 0.0001 | 0.16 | 0.871 | [-0.00019, 0.00022] | < 0.0001 |
| Site RUBIC | 0.0087 | 0.0036 | 2.42 | 0.016 * | [0.00162, 0.01569] | 0.3851 |
| Site SI | 0.0855 | 0.0053 | 16.04 | < 0.001 *** | [0.07500, 0.09592] | 0.3851 |
| Motion | -0.0051 | 0.0025 | -2.02 | 0.044 * | [-0.00999, -0.00013] | 0.0056 |
| ADHD-IN | 0.0071 | 0.0049 | 1.46 | 0.146 | [-0.00248, 0.01673] | 0.0031 |
| ADHD-C | 0.0063 | 0.0049 | 1.27 | 0.206 | [-0.00345, 0.01597] | 0.0031 |

SE: standard error; *f^2^*: Cohen’s f-squared.

# Dimensional Analysis of Full Model

**Model Dim:** FA ~ Age + Sex + IQ + Site + Motion + SWAN-HY

## Whole-Brain

**Table S14:** Dimensional generalized linear mixed effects model (Model Dim) for whole-brain FA.

| **Variable** | **β** | **SE** | **t** | **p** | **95% CI** | **f^2^** |
| --- | --- | --- | --- | --- | --- | --- |
| Age | 0.0016 | 0.0002 | 6.67 | < 0.001 *** | [0.00110, 0.00202] | 0.0617 |
| Sex | 0.0007 | 0.0016 | 0.43 | 0.665 | [-0.00239, 0.00375] | 0.0003 |
| IQ | < -0.0001 | <0.0001 | -0.22 | 0.823 | [-0.00010, 0.00008] | 0.0001 |
| Site RUBIC | 0.0397 | 0.0016 | 25.07 | < 0.001 *** | [0.03655, 0.04276] | 1.9577 |
| Site SI | 0.0834 | 0.0023 | 35.63 | < 0.001 *** | [0.07877, 0.08796] | 1.9577 |
| Motion | -0.0064 | 0.0011 | -5.69 | < 0.001 *** | [-0.00859, -0.00418] | 0.0450 |
| SWAN-HY | -0.0014 | 0.0007 | -1.94 | 0.052 | [-0.00272, 0.00001] | 0.0052 |

SE: standard error; *f^2^*: Cohen’s f-squared.

## Corpus Callosum Forceps Minor

**Table S15:** Dimensional generalized linear mixed effects model (Model Dim) for corpus callosum forceps minor.

| **Variable** | **β** | **SE** | **t** | **p** | **95% CI** | **f^2^** |
| --- | --- | --- | --- | --- | --- | --- |
| Age | 0.0007 | 0.0005 | 1.61 | 0.107 | [-0.00016, 0.00162] | 0.0036 |
| Sex | -0.0002 | 0.0030 | -0.06 | 0.954 | [-0.00611, 0.00576] | < 0.0001 |
| IQ | 0.0002 | 0.0001 | 1.89 | 0.060 | [-0.00001, 0.00034] | 0.0049 |
| Site RUBIC | 0.0563 | 0.0030 | 18.47 | < 0.001 *** | [0.05027, 0.06223] | 0.6171 |
| Site SI | 0.0751 | 0.0045 | 16.63 | < 0.001 *** | [0.06622, 0.08394] | 0.6171 |
| Motion | -0.0140 | 0.0022 | -6.48 | < 0.001 *** | [-0.01824, -0.00976] | 0.0586 |
| SWAN-HY | -0.0025 | 0.0013 | -1.84 | 0.066 | [-0.00511, 0.00016] | 0.0047 |

SE: standard error; *f^2^*: Cohen’s f-squared.

## Corpus Callosum Forceps Major

**Table S16:** Dimensional generalized linear mixed effects model (Model Dim) for corpus callosum forceps major.

| **Variable** | **β** | **SE** | **t** | **p** | **95% CI** | **f^2^** |
| --- | --- | --- | --- | --- | --- | --- |
| Age | 0.0014 | 0.0007 | 1.84 | 0.066 | [-0.00009, 0.00281] | 0.0051 |
| Sex | -0.0068 | 0.0049 | -1.38 | 0.169 | [-0.01642, 0.00288] | 0.0029 |
| IQ | < 0.0001 | 0.0001 | 0.09 | 0.931 | [-0.00027, 0.00029] | < 0.0001 |
| Site RUBIC | 0.0353 | 0.0050 | 7.01 | < 0.001 *** | [0.02543, 0.04523] | 0.1102 |
| Site SI | 0.0538 | 0.0074 | 7.27 | < 0.001 *** | [0.03924, 0.06827] | 0.1102 |
| Motion | -0.0072 | 0.0036 | -1.99 | 0.047 * | [-0.01427, -0.00011] | 0.0060 |
| SWAN-HY | -0.0002 | 0.0022 | -0.10 | 0.923 | [-0.00455, 0.00413] | < 0.0001 |

SE: standard error; *f^2^*: Cohen’s f-squared.

## Left Corticospinal Tract

**Table S17:** Dimensional generalized linear mixed effects model (Model Dim) for left corticospinal tract.

| **Variable** | **β** | **SE** | **t** | **p** | **95% CI** | **f^2^** |
| --- | --- | --- | --- | --- | --- | --- |
| Age | 0.0010 | 0.0005 | 2.23 | 0.026 * | [0.00013, 0.00204] | 0.0071 |
| Sex | 0.0027 | 0.0032 | 0.84 | 0.401 | [-0.00362, 0.00905] | 0.0010 |
| IQ | -0.0001 | 0.0001 | -1.29 | 0.197 | [-0.00031, 0.00006] | 0.0024 |
| Site RUBIC | 0.0757 | 0.0033 | 23.12 | < 0.001 *** | [0.06923, 0.08208] | 1.5776 |
| Site SI | 0.1493 | 0.0048 | 31.13 | < 0.001 *** | [0.13990, 0.15873] | 1.5776 |
| Motion | -0.0036 | 0.0023 | -1.53 | 0.126 | [-0.00811, 0.00100] | 0.0034 |
| SWAN-HY | -0.0030 | 0.0014 | -2.10 | 0.036 * | [-0.00583, -0.00019] | 0.0063 |

SE: standard error; *f^2^*: Cohen’s f-squared.

## Right Corticospinal Tract

**Table S18:** Dimensional generalized linear mixed effects model (Model Dim) for right corticospinal tract.

| **Variable** | **β** | **SE** | **t** | **p** | **95% CI** | **f^2^** |
| --- | --- | --- | --- | --- | --- | --- |
| Age | 0.0007 | 0.0005 | 1.55 | 0.121 | [-0.00020, 0.00169] | 0.0034 |
| Sex | -0.0009 | 0.0032 | -0.27 | 0.788 | [-0.00710, 0.00539] | 0.0001 |
| IQ | -0.0001 | 0.0001 | -1.56 | 0.119 | [-0.00033, 0.00004] | 0.0035 |
| Site RUBIC | 0.0920 | 0.0032 | 28.44 | < 0.001 *** | [0.08564, 0.09834] | 2.0594 |
| Site SI | 0.1643 | 0.0048 | 34.55 | < 0.001 *** | [0.15495, 0.17362] | 2.0594 |
| Motion | -0.0044 | 0.0023 | -1.91 | 0.057 | [-0.00892, 0.00013] | 0.0052 |
| SWAN-HY | -0.0026 | 0.0014 | -1.86 | 0.064 | [-0.00541, 0.00015] | 0.0049 |

SE: standard error; *f^2^*: Cohen’s f-squared.

## Left Superior Longitudinal Fasciculus

**Table S19:** Dimensional generalized linear mixed effects model (Model Dim) for left superior longitudinal fasciculus.

| **Variable** | **β** | **SE** | **t** | **p** | **95% CI** | **f^2^** |
| --- | --- | --- | --- | --- | --- | --- |
| Age | 0.0024 | 0.0005 | 4.35 | < 0.001 *** | [0.00130, 0.00343] | 0.0267 |
| Sex | 0.0067 | 0.0036 | 1.86 | 0.063 | [-0.00038, 0.01383] | 0.0049 |
| IQ | -0.0001 | 0.0001 | -1.22 | 0.224 | [-0.00033, 0.00008] | 0.0021 |
| Site RUBIC | 0.0193 | 0.0037 | 5.27 | < 0.001 *** | [0.01212, 0.02652] | 0.2533 |
| Site SI | 0.0718 | 0.0054 | 13.33 | < 0.001 *** | [0.06125, 0.08241] | 0.2533 |
| Motion | -0.0076 | 0.0026 | -2.91 | 0.004 ** | [-0.01266, -0.00246] | 0.0119 |
| SWAN-HY | -0.0019 | 0.0016 | -1.15 | 0.249 | [-0.00503, 0.00131] | 0.0019 |

SE: standard error; *f^2^*: Cohen’s f-squared.

## Right Superior Longitudinal Fasciculus

**Table S20:** Dimensional generalized linear mixed effects model (Model Dim) for right superior longitudinal fasciculus.

| **Variable** | **β** | **SE** | **t** | **p** | **95% CI** | **f^2^** |
| --- | --- | --- | --- | --- | --- | --- |
| Age | 0.0031 | 0.0005 | 5.95 | < 0.001 *** | [0.00210, 0.00417] | 0.0496 |
| Sex | 0.0038 | 0.0035 | 1.07 | 0.284 | [-0.00313, 0.01067] | 0.0016 |
| IQ | < -0.0001 | 0.0001 | -0.24 | 0.814 | [-0.00023, 0.00018] | 0.0001 |
| Site RUBIC | 0.0089 | 0.0036 | 2.50 | 0.013 * | [0.00190, 0.01588] | 0.3934 |
| Site SI | 0.0847 | 0.0052 | 16.17 | < 0.001 *** | [0.07441, 0.09498] | 0.3934 |
| Motion | -0.0049 | 0.0025 | -1.93 | 0.054 | [-0.00979, 0.00007] | 0.0052 |
| SWAN-HY | 0.0013 | 0.0016 | 0.86 | 0.389 | [-0.00172, 0.00441] | 0.0010 |

SE: standard error; *f^2^*: Cohen’s f-squared.

# Sensitivity Analysis without Excessive Motion

In order to assure that the results obtained are not driven by participants with excessive in-scanner head motion, we repeated the entire analysis on the same sample, yet excluding participants with an average relative motion greater than or equal to 2 mm. A threshold of 2 mm is considered to be a fairly strict excursion criterions, in particular in pediatric studies on mental disorders (compare [20, 56–58]). Within the whole sample 9 participants showed an average relative in-scanner head motion larger or equal to 2 mm, which corresponds to 1.2% of the sample.

For the sample not including participants with average relative in-scanner head motion of greater or equal to 2 mm, all significant results are very similar to the main analysis on the entire sample (see below). We conclude that the main results are not driven by participants with excessive in-scanner head motion.

## Nested Model Comparison

### Categorical Analysis

**Model 1:** Whole-Brain FA ~ Age + Sex + IQ + Site + Motion

**Model 2:** Whole-Brain FA ~ Age + Sex + IQ + Site + Motion + ADHD Category

**Model 3:** Whole-Brain Fa ~ Age + Sex + IQ + Site + Motion * ADHD Category

A comparison of the models using ANOVA yielded the following results:

**Table S21:** Analysis of variance table for categorical analysis using ADHD category and an in-scanner head motion threshold of 2 mm.

|  | **Res.Df** | **RSS** | **Df** | **Sum of Sq** | **F** | **p** |
| --- | --- | --- | --- | --- | --- | --- |
| **Model 1** | 720 | 0.256 |  |  |  |  |
| **Model 2** | 718 | 0.256 | 2 | 0.00040 | 0.56 | 0.573 |
| **Model 3** | 716 | 0.255 | 2 | 0.00103 | 1.45 | 0.235 |

The results are comparable to the analysis with the whole variety of in-scanner head motion. Therefore, we conclude that the identification of the best model is not driven by outliers in average relative in-scanner head motion.

### Dimensional Analysis

**Model 1:** Whole-Brain FA ~ Age + Sex + IQ + Site + Motion

**Model 2:** Whole-Brain FA ~ Age + Sex + IQ + Site + Motion + SWAN-HY

**Model 3:** Whole-Brain Fa ~ Age + Sex + IQ + Site + Motion * SWAN-HY

A comparison of the models using ANOVA yielded the following results:

**Table S22:** Analysis of variance table for dimensional analysis using the SWAN-HY sub-score and an in-scanner head motion threshold of less than 2 mm.

|  | **Res.Df** | **RSS** | **Df** | **Sum of Sq** | **F** | **p** |
| --- | --- | --- | --- | --- | --- | --- |
| **Model 1** | 712 | 0.254 |  |  |  |  |
| **Model 2** | 711 | 0.253 | 1 | 0.00098 | 2.74 | 0.098 |
| **Model 3** | 710 | 0.253 | 1 | 0.00009 | 0.27 | 0.606 |

Also the results for the identification of the best model using a dimensional approach are comparable to the results using the full sample. Consequently, the results were not driven by participants with excessive in-scanner head motion.

### Direct Comparison of Dimensional and Categorical Model

We utilized the same measures as in the main analysis on the entire sample to identify the best model between the dimensional model (Model Dim) and the categorical model (Model Cat) for the subsample without excessive in-scanner head motion.

**Model Cat:** Whole-Brain FA ~ Age + Sex + IQ + Site + Motion + ADHD Category

**Model Dim:** Whole-Brain FA ~ Age + Sex + IQ + Site + Motion + SWAN-HY

The results are summarized in the table below:

**Table S23:** Comparison of measures to assess model fit between dimensional (Model Dim) and categorical model (Model Cat) on subsample with an in-scanner head motion threshold of less than 2 mm.

|  | **Residual SE** | **Multiple R^2^** | **Adjusted R^2^** | **AIC** | **BIC** |
| --- | --- | --- | --- | --- | --- |
| Model Cat | 0.01889 | 0.6963 | 0.6929 | -3656.509 | -3610.731 |
| Model Dim | 0.01886 | 0.6968 | 0.6938 | -3659.625 | -3618.424 |

SE: standard error; AIC: Akaike information criterion; BIC: Bayesian information criterion.

Similarly to the main analysis, in the subsample without excessive in-scanner head motion, the dimensional model (Model Dim) showed smaller AIC and BIC compared to the categorical model (Model Cat).

### Causal Mediation Analysis

We repeated the check for the premise for a causal mediation analysis using the sample excluding participants with an in-scanner head motion threshold of greater or equal to 2 mm. The results are summarized below.

**Table S24:** Summary of regressions of model M0 and model Y as premise for causal mediation analysis according to [59] and [60] with an in-scanner head motion threshold of 2 mm.

| **Structure** | **SWAN-HY → FA** | **SWAN-HY → Motion** |
| --- | --- | --- |
| Whole-Brain FA | *β* = -0.0015  *p* = **0.034 ***  CI = [-0.00288, -0.00011]  *r^2^_SF_ =* 0.00627 | *β* = 0.0574  *p* = **0.010 ****  CI = [0.01381, 0.10106]  *r^2^_SM_* = 0.00930 |
| CC Forceps Minor | *β* = -0.0030  *p* = **0.032 ***  CI = [-0.00566, -0.00026]  *r^2^_SF_ =* 0.00647 | *β* = 0.0553  *p* = **0.013 ***  CI = [0.01147, 0.09909]  *r^2^_SM_ =* 0.00857 |
| CC Forceps Major | *β* = -0.0008  *p* = 0.729  CI = [-0.00514, 0.00360]  *r^2^_SF_* = 0.00018 | *β* = 0.0547  *p* = **0.017 ***  CI = [0.00978, 0.09961]  *r^2^_SM_ =* 0.00870 |
| Left CST | *β* = -0.0032  *p* = **0.028 ***  CI = [-0.00599, -0.00035]  *r^2^_SF_ =* 0.00699 | *β* = 0.0579  *p* = **0.010 ***  CI = [0.01378, 0.10201]  *r^2^_SM_ =* 0.00952 |
| Right CST | *β* = -0.0027  *p* = 0.062  CI = [-0.00546, 0.00013]  *r^2^_SF_ =* 0.00499 | *β* = 0.054488  *p* = **0.015 ***  CI = [0.01080, 0.09818]  *r^2^_SM_ =* 0.00853 |
| Left SLF | *β* = -0.0023  *p* = 0.161  CI = [-0.00549, 0.00091]  *r^2^_SF_ =* 0.00280 | *β* = 0.0635  *p* < **0.005 ****  CI = [0.01955, 0.10751]  *r^2^_SM_ =* 0.01134 |
| Right SLF | *β* = 0.0014  *p* = 0.362  CI = [-0.00165, 0.00452]  *r^2^_SF_ =* 0.00118 | *β* = 0.0570  *p* = **0.011 ***  CI = [0.01307, 0.10096]  *r^2^_SM_ =* 0.00912 |

CC: corpus callosum; CST: corticospinal tract; SLF: superior longitudinal fasciculus; CI: 95% confidence interval; *r^2^_SF_ : portion of variance in FA explained by SWAN-HY; r^2^_SM_ : portion of variance in Motion explained by SWAN-HY.*

Similarly to the full sample, also in the sample without participants with excessive in-scanner head motion the premise for a causal mediation analysis is met for whole-brain FA and average FA of all tracts of interest. As a consequence, we tested the results of the causal mediation analysis on this subsample. The results are summarized in Table S25 below.

**Table S25:** Summary of causal mediation analysis with in-scanner head motion threshold of 2 mm.

| **Structure** | **Average Direct Effect (ADE)** | **Average Causal Mediation Effect (ACME)** |
| --- | --- | --- |
| Whole-Brain FA | *β* = -0.0012  *p* = 0.076  CI = [-0.00243, 0.00016]  *R^2^_direct_ =* 0.00370 | *β* = -0.0003  *p* = **0.006 ****  CI = [-0.00066, -0.00009]  *R^2^_med_* = 0.00256 |
| CC Forceps Minor | *β* = -0.0022  *p* = 0.076  CI = [-0.00473, 0.00027]  *R^2^_direct_ =* 0.00367 | *β* = -0.0007  *p* = **0.011 ***  CI = [-0.00136, -0.00016]  *R^2^_med_* = 0.00279 |
| CC Forceps Major | *β* = -0.0005  *p* = 0.798  CI = [-0.00399, 0.00304]  *R^2^_direct_ =* 0.00007 | *β* = -0.0003  *p* = 0.154  CI = [-0.00092, 0.00011]  *R^2^_med_ =* 0.00012 |
| Left CST | *β* = -0.0030  *p* = **0.027 ***  CI = [-0.00572, -0.00030]  *R^2^_direct_ =* 0.00618 | *β* = -0.0002  *p* = 0.204  CI = [-0.00053, 0.00009]  *R^2^_med_ =* 0.00081 |
| Right CST | *β* = -0.0025  *p* = 0.083  CI = ​​[-0.00523, 0.00029]  *R^2^_direct_ =* 0.00423 | *β* = -0.0002  *p* = 0.145  CI = [-0.00058, 0.00006]  *R^2^_med_ =* 0.00076 |
| Left SLF | *β* = -0.0018  *p* = 0.299  CI = [-0.00520, 0.00158]  *R^2^_direct_ =* 0.00172 | *β* = -0.0005  *p* = **0.005 ****  CI = [-0.00099, -0.00010]  *R^2^_med_ =* 0.00108 |
| Right SLF | *β* = 0.0016  *p* = 0.289  CI = [-0.00141, 0.00466]  *R^2^_direct_ =* 0.00149 | *β* = -0.0002  *p* = 0.242  CI = [-0.00057, 0.00013]  *R^2^_med_ =* - 0.00031 |

CC: corpus callosum; CST: corticospinal tract; SLF: superior longitudinal fasciculus; CI: 95% confidence interval; *R^2^_med_ : portion of variance in FA mediated through ACME; R^2^_direct_ : portion of variance in FA from ADE.*

# Sensitivity Analysis with Average Absolute Displacement

In order to best capture the participants movement behavior, we decided to use the average relative displacement as it is better representing movement over the course of the DTI acquisition, which lasts approximately 10 minutes. Due to occasional use of absolute displacement measures, we have added a sensitivity analysis, reproducing the main analysis using the average absolute displacement as motion measure instead of the average relative displacement. The results are provided below:

## **Nested Model Comparison**

Similarly to the main analysis, we defined the base model as follows:

**Model 1:** Whole-Brain FA ~ Age + Sex + IQ + Site + Absolute Motion

## **Categorical Analysis**

We compared extended models with respect to the base model to identify the benefit of including the ADHD category (categorical, 3 levels) as a fixed effect or an interaction effect between average absolute in-scanner head motion and the ADHD category. Consequently, Model 2 and Model 3 were defined as follows:

**Model 2:** Whole-Brain FA ~ Age + Sex + IQ + Site + Absolute Motion + ADHD Category

**Model 3:** Whole-Brain FA ~ Age + Sex + IQ + Site + Absolute Motion * ADHD Category

A comparison of the models using ANOVA yielded the following results:

**Table S26:** Analysis of variance table for categorical models using the ADHD category.

|  | **Res.Df** | **RSS** | **Df** | **Sum of Sq** | **F** | **p** |
| --- | --- | --- | --- | --- | --- | --- |
| **Model 1** | 729 | 0.266 |  |  |  |  |
| **Model 2** | 729 | 0.266 | 2 | 0.00054 | 0.74 | 0.475 |
| **Model 3** | 725 | 0.265 | 2 | 0.00075 | 1.02 | 0.359 |

In the categorical analysis, none of the extended models including ADHD category as a fixed effect nor as an interaction effect of ADHD category and in-scanner head motion outperformed the base model. Consequently, a categorical approach was not expected to be suitable for causal mediation analysis also when using average absolute displacement instead of average relative displacement.

## **Dimensional Analysis**

In the dimensional analysis we performed the same comparison as in the categorical analysis, but using the SWAN-Hyperactivity sub-score (SWAN-HY; continuous) instead of the ADHD category. The models were defined as follows:

**Model 2:** Whole-Brain FA ~ Age + Sex + IQ + Site + Absolute Motion + SWAN-HY

**Model 3:** Whole-Brain FA ~ Age + Sex + IQ + Site + Absolute Motion * SWAN-HY

A comparison of the models using ANOVA yielded the following results:

**Table S27:** Analysis of variance table for dimensional analysis using the SWAN-Hyperactivity sub-score.

|  | **Res.Df** | **RSS** | **Df** | **Sum of Sq** | **F** | **p** |
| --- | --- | --- | --- | --- | --- | --- |
| **Model 1** | 721 | 0.264 |  |  |  |  |
| **Model 2** | 720 | 0.263 | 1 | 0.00116 | 3.19 | 0.074 |
| **Model 3** | 719 | 0.262 | 1 | 0.00053 | 1.47 | 0.226 |

We concluded to reject a model with an interaction effect between absolute in-scanner head motion and SWAN-HY (Model 3). Although Model 2 turned out only marginally better in explaining the variance in the data than the base model, we decided to include SWAN-HY as a main effect since it is a variable of interest and would match the model specification in the main analysis.

Therefore, we concluded to proceed with the following final model design:

**Model Dim:** FA ~ Age + Sex + IQ + Site + Motion + SWAN-HY

### **Causal Mediation Analysis**

Before computing the mediation, we verified the premise for mediation in accordance to [59] and [60]. The results are summarized below.

**Table S28:** Summary of regressions of model M0 and model Y as premise for causal mediation analysis according to [59] and [60] with average absolute displacement as measure for in-scanner head motion.

| **Structure** | **SWAN-HY → FA** | **SWAN-HY → Motion** |
| --- | --- | --- |
| Whole-Brain FA | *β* = -0.0017  *p* = **0.015 ***  CI = [-0.00311, -0.00033]  *r^2^_SF_* = 0.00814 | *β* = -0.0005  *p* < **0.001 *****  CI = [-0.00616, -0.00286]  *r^2^_SM_* = 0.01572 |
| CC Forceps Minor | *β* = -0.0033  *p* = **0.018 ***  CI = [-0.00595, -0.00056]  *r^2^_SF_* = 0.00774 | *β* = -0.0100  *p* < **0.001 *****  CI = [-0.01321, -0.00685]  *r^2^_SM_* = 0.01478 |
| CC Forceps Major | *β* = -0.0006  *p* = 0.790  CI = [-0.00492, 0.00375]  *r^2^_SF_* = 0.00011 | *β* = -0.0049  *p* = 0.065  CI = [-0.01019, 0.00030]  *r^2^_SM_* = 0.01224 |
| Left CST | *β* = -0.0032  *p* = **0.025 ***  CI = [-0.00602, -0.00041]  *r^2^_SF_* = 0.00717 | *β* = -0.0035  *p* = **0.0443 ***  CI = [-0.00688, -0.00009]  *r^2^_SM_* = 0.01557 |
| Right CST | *β* = -0.0029  *p* = **0.043 ***  CI = [-0.00565, -0.00010]  *r^2^_SF_* = 0.00581 | *β* = -0.0042  *p* = **0.015 ***  CI = [-0.00759, -0.00083]  *r^2^_SM_* = 0.01548 |
| Left SLF | *β* = -0.0023  *p* = 0.148  CI = [-0.00551, 0.00083]  *r^2^_SF_* = 0.00295 | *β* = -0.0033  *p* = 0.095  CI = [-0.00710, 0.00057]  *r^2^_SM_* = 0.01736 |
| Right SLF | *β* = 0.0011  *p* = 0.494  CI = [-0.00199, 0.00412]  *r^2^_SF_* = 0.00066 | *β* = -0.0024  *p* = 0.198  CI = [-0.00614, 0.00128]  *r^2^_SM_* = 0.01516 |

CC: corpus callosum; CST: corticospinal tract; SLF: superior longitudinal fasciculus; CI: 95% confidence interval; r^2^_SF_ : portion of variance in FA explained by SWAN-HY; r^2^_SM_ : portion of variance in Motion explained by SWAN-HY.

Comparable to the main analysis, also in this analysis using average absolute displacement the premise for a causal mediation analysis is met for whole-brain FA and most tracts of interest. For reasons of completeness and within the scope of the sensitivity analysis, although the premise was not met for all structures, we tested the results of the causal mediation analysis. The results are summarized in Table S29 below.

**Table S29:** Summary of causal mediation analysis with average absolute displacement as measure for in-scanner head motion.

| **Structure** | **Average Direct Effect (ADE)** | **Average Causal Mediation Effect (ACME)** |
| --- | --- | --- |
| Whole-Brain FA | *β* = -0.0013  *p* = 0.067  CI = [-0.00266, 0.00008]  *R^2^_direct_* = 0.00423 | *β* = -0.0005  *p* < **0.001 *****  CI = [-0.00085, -0.00017]  *R^2^_med_* = 0.00391 |
| CC Forceps Minor | *β* = -0.0022  *p* = 0.092  CI = [-0.00483, 0.00032]  *R^2^_direct_* = 0.00361 | *β* = -0.0010  *p* = **0.002 ****  CI = [-0.00181, -0.00037]  *R^2^_med_* = 0.00413 |
| CC Forceps Major | *β* = -0.0001  *p* = 0.950  CI = [-0.00371, 0.00349]  *R^2^_direct_* = 0.00001 | *β* = -0.0005  *p* = 0.070  CI = [-0.00116, 0.00003]  *R^2^_med_* = 0.00010 |
| Left CST | β = -0.0029  *p* = **0.041 ***  CI = [-0.00564, -0.00010]  *R^2^_direct_* = 0.00556 | *β* = -0.0004  *p* = 0.058  CI = [-0.00089, 0.00001]  *R^2^_med_* = 0.00161 |
| Right CST | *β* = -0.0024  *p* = 0.095  CI = [-0.00525, 0.00044]  *R^2^_direct_* = 0.00413 | *β* = -0.0004  *p* = **0.020 ***  CI = [-0.00093, -0.00005]  *R^2^_med_* = 0.00168 |
| Left SLF | *β* = -0.0020  *p* = 0.226  CI = [-0.00530, 0.00125]  *R^2^_direct_* = 0.00208 | *β* = -0.0004  *p* = 0.096  CI = [-0.00088, 0.00005]  *R^2^_med_* = 0.00087 |
| Right SLF | *β* = 0.0013  *p* = 0.396  CI = [-0.00167, 0.00446]  *R^2^_direct_* = 0.00098 | *β* = -0.0002  *p* = 0.201  CI = [-0.00073, 0.00013]  *R^2^_med_* = -0.00033 |

CC: corpus callosum; CST: corticospinal tract; SLF: superior longitudinal fasciculus; CI: 95% confidence interval; R^2^_med_ : portion of variance in FA mediated through ACME; R^2^_direct_ : portion of variance in FA from ADE.

# Calculation of effective number of variables (M_eff_)

In order to evaluate the results also at a corrected significance level, the effective number of variables for correction of multiple testing was computed according to Nyholt (2004) [61]. The eigenvalues of the observations were calculated based on the six tracts of interest. The variance of the eigenvalues of the observations were estimated as:

$$Var\left( \lambda_{obs} \right)= 1.3042$$

The true number of variables (i.e. the number of tracts) was set to:

$$M=6$$

According to the formula from [61]:

$$M_{eff}= 1+ \left( M-1 \right)\left( 1-\frac{Var\left( \lambda_{obs} \right)}{M} \right)$$

the effective number of variables was estimated as:

$$M_{eff}= 4.91$$

Consequently, results should be considered as surviving correction for multiple testing at a significance level of:

$$p< \frac{0.05}{4.91}=0.0102$$

# References

1. Chou KH, Chou YH, Chiu FY, Gao WY, Su, TP, Chu WC *et al.* Structural abnormalities of adolescent males with attention-deficit/hyperactivity disorder: a DTI study. In *The joint Annual Meeting ISMRM-ESMRMB* 2007; **1**:8-16.
2. Silk TJ, Vance A, Rinehart N, Bradshaw JL, Cunnington R. White-matter abnormalities in attention deficit hyperactivity disorder: A diffusion tensor imaging study. *Human Brain Mapping* 2009; **30**: 2757–2765.
3. Francx W, Llera A, Mennes M, Zwiers MP, Faraone SV, Oosterlaan J, *et al.* Integrated analysis of gray and white matter alterations in attention-deficit/hyperactivity disorder. *Neuroimage Clin* 2016; **11**: 357–367.
4. Cao Q, Sun L, Gong G, Lv Y, Cao X, Shuai L, *et al.* The macrostructural and microstructural abnormalities of corpus callosum in children with attention deficit/hyperactivity disorder: a combined morphometric and diffusion tensor MRI study. *Brain Res* 2010; **1310**: 172–180.
5. Qiu M-G, Ye Z, Li Q-Y, Liu G-J, Xie B, Wang J. Changes of brain structure and function in ADHD children. *Brain Topogr* 2011; **24**: 243–252.
6. Chuang T-C, Wu M-T, Huang S-P, Weng M-J, Yang P. Diffusion tensor imaging study of white matter fiber tracts in adolescent attention-deficit/hyperactivity disorder. *Psychiatry Res* 2013; **211**: 186–187.
7. van Ewijk H, Heslenfeld DJ, Zwiers MP, Faraone SV, Luman M, Hartman CA, *et al.* Different mechanisms of white matter abnormalities in attention-deficit/hyperactivity disorder: a diffusion tensor imaging study. *J Am Acad Child Adolesc Psychiatry* 2014; **53**: 790–9.e3.
8. Langevin LM, Macmaster FP, Crawford S, Lebel C, Dewey D. Common white matter microstructure alterations in pediatric motor and attention disorders. *J Pediatr* 2014; **164**: 1157–1164.e1.
9. Pastura G, Doering T, Gasparetto EL, Mattos P, Araújo AP. Exploratory analysis of diffusion tensor imaging in children with attention deficit hyperactivity disorder: evidence of abnormal white matter structure. *Atten Defic Hyperact Disord* 2016; **8**: 65–71.
10. Ameis SH, Lerch JP, Taylor MJ, Lee W, Viviano JD, Pipitone J, *et al.* A Diffusion Tensor Imaging Study in Children With ADHD, Autism Spectrum Disorder, OCD, and Matched Controls: Distinct and Non-Distinct White Matter Disruption and Dimensional Brain-Behavior Relationships. *Am J Psychiatry* 2016; **173**: 1213–1222.
11. Wu Z-M, Bralten J, Cao Q-J, Hoogman M, Zwiers MP, An L, *et al.* White Matter Microstructural Alterations in Children with ADHD: Categorical and Dimensional Perspectives. *Neuropsychopharmacology* 2017; **42**: 572–580.
12. Lin Q, Bu X, Wang M, Liang Y, Chen H, Wang W, *et al.* Aberrant white matter properties of the callosal tracts implicated in girls with attention-deficit/hyperactivity disorder. *Brain Imaging Behav* 2020; **14**: 728–735.
13. Bu X, Yang C, Liang K, Lin Q, Lu L, Zhang L, *et al.* Quantitative tractography reveals changes in the corticospinal tract in drug-naïve children with attention-deficit/hyperactivity disorder. *J Psychiatry Neurosci* 2020; **45**: 134–141.
14. Pavuluri MN, Yang S, Kamineni K, Passarotti AM, Srinivasan G, Harral EM, *et al.* Diffusion tensor imaging study of white matter fiber tracts in pediatric bipolar disorder and attention-deficit/hyperactivity disorder. *Biol Psychiatry* 2009; **65**: 586–593.
15. Svatkova A, Nestrasil I, Rudser K, Goldenring Fine J, Bledsoe J, Semrud-Clikeman M. Unique white matter microstructural patterns in ADHD presentations-a diffusion tensor imaging study. *Hum Brain Mapp* 2016; **37**: 3323–3336.
16. Ashtari M, Kumra S, Bhaskar SL, Clarke T, Thaden E, Cervellione KL, *et al.* Attention-deficit/hyperactivity disorder: a preliminary diffusion tensor imaging study. *Biol Psychiatry* 2005; **57**: 448–455.
17. Hamilton LS, Levitt JG, O’Neill J, Alger JR, Luders E, Phillips OR, *et al.* Reduced white matter integrity in attention-deficit hyperactivity disorder. *Neuroreport* 2008; **19**: 1705–1708.
18. Bechtel N, Kobel M, Penner I-K, Klarhöfer M, Scheffler K, Opwis K, Weber P. Decreased fractional anisotropy in the middle cerebellar peduncle in children with epilepsy and/or attention deficit/hyperactivity disorder: a preliminary study. *Epilepsy Behav* 2009; **15**: 294–298.
19. Kobel M, Bechtel N, Specht K, Klarhöfer M, Weber P, Scheffler K, *et al.* Structural and functional imaging approaches in attention deficit/hyperactivity disorder: does the temporal lobe play a key role? *Psychiatry Res* 2010; **183**: 230–236.
20. Nagel BJ, Bathula D, Herting M, Schmitt C, Kroenke CD, Fair D, Nigg JT. Altered white matter microstructure in children with attention-deficit/hyperactivity disorder. *J Am Acad Child Adolesc Psychiatry* 2011; **50**: 283–292.
21. Chiang H-L, Chen Y-J, Lo Y-C, Tseng W-YI, Gau SS. Altered white matter tract property related to impaired focused attention, sustained attention, cognitive impulsivity and vigilance in attention-deficit/ hyperactivity disorder. *J Psychiatry Neurosci* 2015; **40**: 325–335.
22. Chiang H-L, Chen Y-J, Shang C-Y, Tseng W-YI, Gau SS-F. Different neural substrates for executive functions in youths with ADHD: a diffusion spectrum imaging tractography study. *Psychol Med* 2016; **46**: 1225–1238.
23. Davenport ND, Karatekin C, White T, Lim KO. Differential fractional anisotropy abnormalities in adolescents with ADHD or schizophrenia. *Psychiatry Research: Neuroimaging*, 2010; **181**: 193–198.
24. Tamm L, Barnea-Goraly N, Reiss AL. Diffusion tensor imaging reveals white matter abnormalities in Attention-Deficit/Hyperactivity Disorder. *Psychiatry Res* 2012; **202**: 150–154.
25. Peterson DJ, Ryan M, Rimrodt SL, Cutting LE, Denckla MB, Kaufmann WE, Mahone EM. Increased regional fractional anisotropy in highly screened attention-deficit hyperactivity disorder (ADHD). *J Child Neurol* 2011; **26**: 1296–1302.
26. Alexander LM, Escalera J, Ai L, Andreotti C, Febre K, Mangone A, *et al.* An open resource for transdiagnostic research in pediatric mental health and learning disorders. *Sci Data* 2017; **4**: 170181.
27. Veraart J, Fieremans E, Novikov DS. Diffusion MRI noise mapping using random matrix theory. *Magn Reson Med* 2016; **76**: 1582–1593.
28. Kellner E, Dhital B, Kiselev VG, Reisert M. Gibbs-ringing artifact removal based on local subvoxel-shifts. *Magn Reson Med* 2016; **76**: 1574–1581.
29. Studholme C, Constable RT, Duncan JS. Accurate alignment of functional EPI data to anatomical MRI using a physics-based distortion model. *IEEE Trans Med Imaging* 2000; **19**: 1115–1127.
30. Jenkinson M, Beckmann CF, Behrens TEJ, Woolrich MW, Smith SM. FSL. *Neuroimage* 2012; **62**: 782–790.
31. Smith SM. Fast robust automated brain extraction. *Hum Brain Mapp* 2002; **17**: 143–155.
32. Andersson JLR, Sotiropoulos SN. An integrated approach to correction for off-resonance effects and subject movement in diffusion MR imaging. *Neuroimage* 2016; **125**: 1063–1078.
33. Andersson JLR, Graham MS, Zsoldos E, Sotiropoulos SN. Incorporating outlier detection and replacement into a non-parametric framework for movement and distortion correction of diffusion MR images. *Neuroimage* 2016; **141**: 556–572.
34. Andersson JLR, Graham MS, Drobnjak I, Zhang H, Filippini N, Bastiani M. Towards a comprehensive framework for movement and distortion correction of diffusion MR images: Within volume movement. *Neuroimage* 2017; **152**: 450–466.
35. Aoki Y, Cortese S, Castellanos FX. Research Review: Diffusion tensor imaging studies of attention-deficit/hyperactivity disorder: meta-analyses and reflections on head motion. *J Child Psychol Psychiatry* 2018; **59**: 193–202.
36. Yendiki A, Koldewyn K, Kakunoori S, Kanwisher N, Fischl B. Spurious group differences due to head motion in a diffusion MRI study. *NeuroImage*, 2014; **88**: 79–90.
37. Tijssen RHN, Jansen JFA, Backes WH. Assessing and minimizing the effects of noise and motion in clinical DTI at 3 T. *Hum Brain Mapp* 2009; **30**: 2641–2655.
38. Collier Q, Veraart J, Jeurissen B, den Dekker AJ, Sijbers J. Iterative reweighted linear least squares for accurate, fast, and robust estimation of diffusion magnetic resonance parameters. *Magn Reson Med* 2015; **73**: 2174–2184.
39. Veraart J, Sijbers J, Sunaert S, Leemans A, Jeurissen B. Weighted linear least squares estimation of diffusion MRI parameters: strengths, limitations, and pitfalls. *Neuroimage* 2013; **81**: 335–346.
40. Veraart J, Poot DHJ, Van Hecke W, Blockx I, Van der Linden A, Verhoye M, Sijbers J. More accurate estimation of diffusion tensor parameters using diffusion Kurtosis imaging. *Magn Reson Med* 2011; **65**: 138–145.
41. Fieremans E, Jensen JH, Helpern JA. White matter characterization with diffusional kurtosis imaging. *Neuroimage* 2011; **58**: 177–188.
42. Yeatman JD, Dougherty RF, Myall NJ, Wandell BA, Feldman HM. Tract profiles of white matter properties: automating fiber-tract quantification. *PLoS One* 2012; **7**: e49790.
43. Mori S, Crain BJ, Chacko VP, van Zijl PC. Three-dimensional tracking of axonal projections in the brain by magnetic resonance imaging. *Ann Neurol* 1999; **45**: 265–269.
44. Basser PJ, Pajevic S, Pierpaoli C, Duda J, Aldroubi A. In vivo fiber tractography using DT-MRI data. *Magnetic Resonance in Medicine* 2000; **44**: 625–632.
45. Wassermann D, Rathi Y, Bouix S, Kubicki M, Kikinis R, Shenton M, Westin C-F. White matter bundle registration and population analysis based on Gaussian processes. *Inf Process Med Imaging* 2011; **22**: 320–332.
46. Yeatman JD, Dougherty RF, Rykhlevskaia E, Sherbondy AJ, Deutsch GK, Wandell BA, Ben-Shachar M. Anatomical properties of the arcuate fasciculus predict phonological and reading skills in children. *J Cogn Neurosci* 2011; **23**: 3304–3317.
47. Wakana S, Caprihan A, Panzenboeck MM, Fallon JH, Perry M, Gollub RL, *et al.* Reproducibility of quantitative tractography methods applied to cerebral white matter. *Neuroimage* 2007; **36**: 630–644.
48. Chen L, Hu X, Ouyang L, He N, Liao Y, Liu Q, *et al.* A systematic review and meta-analysis of tract-based spatial statistics studies regarding attention-deficit/hyperactivity disorder. *Neurosci Biobehav Rev* 2016; **68**: 838–847.
49. van Ewijk H, Heslenfeld DJ, Zwiers MP, Buitelaar JK, Oosterlaan J. Diffusion tensor imaging in attention deficit/hyperactivity disorder: a systematic review and meta-analysis. *Neurosci Biobehav Rev* 2012; **36**: 1093–1106.
50. Beaulieu C. The basis of anisotropic water diffusion in the nervous system - a technical review. *NMR Biomed* 2002; **15**: 435–455.
51. Jones DK, Knösche TR, Turner R. White matter integrity, fiber count, and other fallacies: the do’s and don'ts of diffusion MRI. *Neuroimage* 2013; **73**: 239–254.
52. Mädler B, Drabycz SA, Kolind SH, Whittall KP, MacKay AL. Is diffusion anisotropy an accurate monitor of myelination? Correlation of multicomponent T2 relaxation and diffusion tensor anisotropy in human brain. *Magn Reson Imaging* 2008; **26**: 874–888.
53. Ades-Aron B, Veraart J, Kochunov P, McGuire S, Sherman P, Kellner E, *et al.* Evaluation of the accuracy and precision of the diffusion parameter EStImation with Gibbs and NoisE removal pipeline. *Neuroimage* 2018; **183**: 532–543.
54. Andersson JL, Skare S, Ashburner J. How to correct susceptibility distortions in spin-echo echo-planar images: application to diffusion tensor imaging. *Neuroimage* 2003; **20(2)**: 870-888.
55. Wilkinson GN, Rogers CE. Symbolic description of factorial models for analysis of variance. *Journal of the Royal Statistical Society: Series C (Applied Statistics)* 1973; **22(3)**: 392-399.
56. Yoncheva YN, Somandepalli K, Reiss PT, Kelly C, Di Martino A, Lazar M, *et al.* Mode of Anisotropy Reveals Global Diffusion Alterations in Attention-Deficit/Hyperactivity Disorder. *J Am Acad Child Adolesc Psychiatry* 2016; **55**: 137–145.
57. Aoki Y, Yoncheva YN, Chen B, Nath T, Sharp D, Lazar M, *et al.* Association of White Matter Structure With Autism Spectrum Disorder and Attention-Deficit/Hyperactivity Disorder. *JAMA Psychiatry* 2017; **74**: 1120–1128.
58. Bessette KL, Stevens MC. Neurocognitive Pathways in Attention-Deficit/Hyperactivity Disorder and White Matter Microstructure. *Biol Psychiatry Cogn Neurosci Neuroimaging* 2019; **4**: 233–242.
59. Baron RM, Kenny DA. The moderator-mediator variable distinction in social psychological research: conceptual, strategic, and statistical considerations. *J Pers Soc Psychol* 1986; **51**: 1173–1182.
60. Shrout PE, Bolger N. Mediation in experimental and nonexperimental studies: new procedures and recommendations. *Psychol Methods* 2002; **7**: 422–445.
61. Nyholt DR. A simple correction for multiple testing for single-nucleotide polymorphisms in linkage disequilibrium with each other. *Am J Hum Genet* 2004; **74**: 765–769.
